# A hybrid framework for disease biomarker discovery in microbiome research combining Bayesian networks, machine learning, and network-based methods

**DOI:** 10.1093/biomethods/bpaf089

**Published:** 2025-12-13

**Authors:** Rosa Aghdam, Shan Shan, Richard Lankau, Claudia Solís-Lemus

**Affiliations:** Wisconsin Institute for Discovery, University of Wisconsin-Madison, Madison, WI 53715, United States; Department of Plant Pathology, University of Wisconsin-Madison, Madison, WI 53715, United States; Department of Plant Pathology, University of Wisconsin-Madison, Madison, WI 53715, United States; Wisconsin Institute for Discovery, University of Wisconsin-Madison, Madison, WI 53715, United States; Department of Plant Pathology, University of Wisconsin-Madison, Madison, WI 53715, United States

**Keywords:** Bayesian networks, bootstrap analysis, microbiome network, soil microbiome, CMIMN R package, multi-method feature selection

## Abstract

Microbiome research faces two central challenges, namely constructing reliable networks, where nodes represent microbial taxa and edges represent their associations, and identifying significant disease-associated taxa. To address the first challenge, we developed CMIMN, a novel R package that applies a Bayesian network framework based on conditional mutual information to infer microbial interaction networks. To further enhance reliability, we construct a consensus microbiome network by integrating results from CMIMN and three widely used methods, including Sparse Inverse Covariance Estimation for Ecological Association Inference (SPIEC-EASI), Semi-Parametric Rank-based correlation and partial correlation Estimation (SPRING), and Sparse Correlations for Compositional Data (SPARCC). This consensus approach, which overlays and weights edges shared across methods, reduces inconsistencies and provides a more biologically meaningful view of microbial relationships. To address the second challenge, we designed a multi-method feature selection framework that combines machine learning with network-based strategies. Our machine learning pipeline applies distinct algorithms and identifies key taxa based on their consistent importance across models. Complementing this, we employ two network-based strategies that prioritize taxa based on centrality differences between networks constructed from healthy samples and disease-affected samples, as well as a composite scoring system that ranks nodes using integrated network metrics. We applied CMIMN on soil microbiome data from potato fields affected by common scab disease. Bootstrap analysis confirmed the robustness of CMIMN, and the consensus network further improved stability and interpretability. The multi-method framework enhances confidence in identifying soil microbial taxa associated with potato disease. Notably, we identified *Bacteroidota*, *WPS-2*, and *Proteobacteria* at the Phylum level; *Actinobacteria*, *AD3*, *Bacilli*, *Anaerolineae*, and *Ktedonobacteria* at the Class level; and *C0119*, *Defluviicoccales*, *Bacteroidales*, and *Ktedonobacterales* at the Order level as key taxa associated with disease status.

## Introduction

Microbial communities play critical roles in human, animal, and plant health, yet their complexity poses significant challenges for understanding underlying interactions [1–11]. This complexity arises from several sources, including the high diversity of bacteria, archaea, fungi, and microeukaryotes that coexist within these systems; the broad spectrum of ecological relationships among them ranging from mutualism and commensalism to competition and predation; and the intricate network connectivity characterized by dense interactions, modular organization, and influential keystone taxa that maintain community stability [12]. In ecological theory, the link between complexity and stability has been debated for decades: while some studies suggest that greater diversity and interaction richness stabilize ecosystems, others propose that excessive complexity can amplify fragility. Microbiomes offer an ideal system to explore this relationship because they are among the most complex biological communities on Earth [13] encompassing thousands of taxa, spanning all major domains of life, and exhibiting the full range of positive and negative interactions across virtually every environment [4, 14, 15]. Environmental heterogeneity and the compositional nature of sequencing data further contribute to this complexity, making microbiome network inference a uniquely challenging yet powerful approach to understanding ecosystem stability [16].

One common strategy is to construct microbiome networks, where taxa are represented as nodes and their associations as edges; this perspective offers a rigorous framework for characterizing complex interactions and has been applied widely across ecological and molecular systems [17]. Well-known inference methods for this purpose include Sparse Correlations for Compositional Data (SPARCC) [18], SParse InversE Covariance Estimation for Ecological Association Inference (SPIEC-EASI) [19], and the Semi-Parametric Rank-based approach for INference in Graphical models (SPRING) [20]. These methods have provided important tools for exploring microbial associations and have yielded insights into community structure and functional relationships [21]. However, they often produce inconsistent results, with different algorithms generating markedly different network structures from the same dataset [17]. Moreover, there is no universally accepted gold standard for evaluating inferred microbiome networks, which makes it difficult to assess the reliability of associations. In related fields such as gene regulatory network inference, benchmarking is possible because experimentally validated gold standard datasets exist, allowing direct comparison between inferred and true networks [22–24]. In microbiome research, however, such validated interaction maps are not available, making it particularly challenging to rigorously evaluate and compare new inference algorithms. Consequently, developing novel and more robust algorithms for microbiome network construction has become a highly active area of research. At the same time, there is also a need for consensus-driven frameworks that integrate multiple inference strategies to improve reproducibility and reliability [25].

In addition to challenges in network construction, microbiome data itself is difficult to analyze because it is sparse, high-dimensional, and compositional [2]. Another central challenge is the reliable identification of disease-associated taxa, which is essential for understanding disease mechanisms and developing actionable strategies to improve host health and productivity [2, 26]. Three main classes of methods are commonly used for this purpose. First, machine learning (ML) approaches (e.g., random forests and gradient boosting) identify taxa that are predictive of disease outcomes [2]. Second, statistical differential abundance tests, including ANalysis of Differential Abundance using Compositional data (ALDEx2) [27], ANalysis of Composition of Microbiomes with Bias Correction (ANCOM-BC) [28], COunt RegressioN for Correlated Observations with the Beta-binomial (corncob) [29], and LINear Models for Differential Abundance (LinDA) [30], compare microbial abundances between conditions [27, 28, 30–32]. Third, network-based inference methods use structural properties, such as centrality, to highlight taxa that may act as keystones [25, 33]. Because these strategies rely on different methods and underlying assumptions, they often identify distinct sets of taxa, leading to inconsistent conclusions across studies. Developing integrative frameworks is therefore critical for obtaining robust and biologically meaningful insights.

This study builds upon prior research that explored associations between soil properties and biological phenotypes using ML models, including random forests and Bayesian neural networks [2]. Extending this work, we adopt a network-based perspective, specifically leveraging Bayesian networks (BNs), to investigate microbial relationships within the soil microbiome. To enhance the robustness of disease-associated operational taxonomic units identification, we introduce a comprehensive multi-method framework that combines ML-based feature selection with network-based strategies. This integrative design ensures that candidate taxa are supported by both predictive modeling and ecological network context, thereby increasing confidence in their biological relevance. The main contributions of this study are as follows: (1) We present a novel BN-based algorithm, Conditional Mutual Information (CMI)-based Microbiome Network (CMIMN), implemented in the R package CMIMN; (2) We propose a consensus network approach that integrates results from CMIMN, SPIEC-EASI, SPRING, and SPARCC, improving the reliability of inferred microbial associations; and (3) We develop a dual feature selection strategy that incorporates both ML outputs and network centrality metrics to identify key disease-associated taxa. Importantly, the methodology introduced here is broadly applicable and can be adapted for analyzing disease-related microbiome datasets across both agricultural and clinical domains.

To illustrate the applicability of our framework, we apply it to the soil microbiome of potato fields affected by common scab disease as a representative case study. Potatoes, as the world’s fourth most essential crop, play a vital role in addressing global food security. However, soil-borne diseases like potato common scab, caused by *Streptomyces* scabies and related pathogens, significantly threaten potato yield and quality. This disease results in economic losses due to the rejection of tubers with pitted, corky lesions and has broader implications for food security. While some growers and consultants may claim that fumigation with broad-spectrum fungicides controls common scab symptoms, evidence for its effectiveness remains inconsistent, and fumigation is often costly and environmentally restrictive [34]. In practice, genetic resistance (or tolerance) in potato cultivars has emerged as a more effective and sustainable strategy for managing the disease [35]. A promising complementary approach involves exploiting the potential of naturally disease-suppressive soils, which harbor specific microbial communities that suppress pathogens and reduce disease outbreaks. Research has shown that suppressive soils often harbor distinct microbial communities with a higher abundance of antagonistic taxa, such as non-pathogenic *Streptomyces spp.* and *Bacillus* [36, 37]. These insights underscore the importance of investigating microbial interactions in suppressive soils to guide environmentally sustainable disease control practices. This dataset provides an ideal benchmark for evaluating the robustness, interpretability, and biological relevance of the CMIMN algorithm and the consensus-based strategy. By constructing microbial networks and applying our multi-method feature selection framework, we identify key taxa linked to disease resistance and highlight the potential of this approach for uncovering reproducible and biologically meaningful microbial associations.

## Materials and methods

### Data preparation

In this study, we focus on the soil microbiome (matrix of abundances) in a variety of taxonomic orders, including Phylum, Class, and Order from soil samples acquired from potato fields in Wisconsin and Minnesota. We concentrated on these three taxonomic levels due to their balance of interpretability and feature dimensionality, allowing for a meaningful analysis of microbial community structure and its association with disease. The dataset consists of microbial community data of pre-planting soils and the corresponding disease levels in the plants at harvest. Overall, we collected 256 soil samples, 108 of which were taken from 36 commercial fields in Minnesota, and 148 of which were taken from 50 fields in Wisconsin. This extensive dataset provides a comprehensive representation of soil microbial communities across two major potato-growing regions in the Upper Midwest. To obtain the microbiome data, DNA was extracted from 0.25 g of pre-planting soils using the DNeasy PowerSoil Pro DNA isolation kit (Qiagen, CA) following the manufacturer’s instructions. Extracted DNA was used in a two-step polymerase chain reaction where the V3-V4 region of bacterial 16S rRNA and eukaryotic ITS2 region were amplified for sequencing on an Illumina MiSeq platform at the University of Minnesota Genomics Center. Sequence data was processed following a pipeline developed in Lankau lab [2]. Sequences were trimmed, quality filtered, denoised, pair-end merged, and chimeras removed. Taxonomy was assigned to the feature table of amplicon sequence variant using the Ribosomal Database Project (RDP) Naïve Bayesian Classifier fit to the SILVA 138 database for 16S reads and UNITE database for Internal Transcribed Spacer (ITS) reads. Bacterial and fungal amplicon sequence variant tables were merged at the Phylum, Class, and Order levels using phyloseq in R. Merged taxa at these higher taxonomic levels were referred to as “OTU” in this article. At harvest, potatoes were hand-harvested from a one-meter hill (usually 3–4 plants) at each sampling location. Tubers from one plant were visually evaluated for the presence of pitted scab lesions: which is a sign for serious common scab disease, as these tubers would be excluded from marketable yield. This binary disease label, 0 for healthy and 1 for diseased, serves as the target variable in our analyses, linking microbial community features to agricultural outcomes. The input data is a matrix with nonnegative read counts that were generated by a sequencing procedure, filtered out so that we only include Operational Taxonomic Units (OTUs) that appear in at least 15 samples [2]. This filtering ensures a focus on microbial taxa with sufficient prevalence to contribute meaningfully to statistical and network-based analyses, reducing noise from rare taxa. Supplementary Table S3 displays the number of features (OTUs) before and after filtering for different taxonomic levels. To enhance reproducibility and facilitate further research, the raw sequencing data and preprocessing scripts are available upon request or through the project repository.

### Constructing microbiome networks

#### CMI algorithm for constructing microbiome networks

We outline the methodology behind the CMIMN algorithm, a novel approach for constructing microbiome networks. First, we introduce the foundational concepts of MI and CMI, which are key components of the CMIMN framework. Next, we provide an overview of BNs, their structure, and their applicability to microbiome research. Finally, we describe the detailed steps of the CMIMN algorithm, highlighting its dynamic thresholding and order-independent features that address the unique challenges of microbiome datasets.

##### MI and CMI

MI and CMI are proven to be effective for detecting relationships between variables due to their capability to measure nonlinear dependencies [38]. In the context of microbiome data, these variables correspond to microbial taxa, where *X* and *Y* represent the abundance profiles of two taxa (e.g., two OTUs), and **Z** denotes a set of other taxa whose effects are conditioned upon. MI and CMI between the variables *X* and *Y*, given the vector of variables **Z**, are defined as follows [39]:


(1) $$MI(X,Y)=\int_{R}\int_{R}p(x,y)\;\;\text{log}\;\frac{p(x,y)}{p(x)\;p(y)}\;dx\;dy,$$

(2) $$\mathrm{CMI}(X,Y|Z)=\int_{R^{p}}\int_{R}\int_{R}p(x,y,z)\;\;\text{log}\;\frac{p(x,y|z)}{p(x|z)\;p(y|z)}\;dx\;dy\;dz$$

where *p* is the dimension of vector **Z** and p(x,y), p(x) and p(y) represent the joint distribution of the taxa *X* and *Y*, marginal distribution of *X*, marginal distribution of *Y*, respectively. p(x,y,z), p(x,y|z), p(x|z) and p(y|z) indicate joint distribution of *X*, *Y* and **Z**, the conditional density distribution of *X* and *Y* given **Z**, the conditional density distribution of *X* given **Z** and the conditional density distribution of *Y* given **Z**, respectively. Under the assumption that data follows a Gaussian distribution, MI for two continuous variables *X* and *Y* can be calculated as [22, 40, 41]:


(3) $$MI(X,Y)=\frac{1}{2}\text{log}\;\frac{\sigma_{X}^{2}\sigma_{Y}^{2}}{\sigma_{XY}},$$

where $\sigma_{X}^{2}$, $\sigma_{Y}^{2}$ and $\sigma_{XY}$ indicate the variance of *X*, the variance of *Y* and the covariance between *X* and *Y*, respectively. When *X* and *Y* are independent, then MI(X,Y)=0. Similarly, CMI(X,Y|Z) is defined as:


(4) $$\mathrm{CMI}(X,Y|Z)=\frac{1}{2}\text{log}\;\frac{\left|C(X,Z)||C(Y,Z)\right|}{\left|C(Z)||C(X,Y,Z)\right|},$$

where C is the covariance matrix and |.| is the determinant of matrix C. C(X, Y) and C(X, Y, **Z**) denote the covariance matrix of variables *X* and *Y* and variables X, Y, and **Z**, respectively. When *X* and *Y* are conditionally independent given **Z**, then CMI(X,Y|Z)=0. These measures form the backbone of many network inference methods, including BNs, which are particularly suited for capturing complex dependencies in microbiome datasets. Biologically, MI quantifies how much knowing the abundance of one microbial taxon (e.g., *X*) reduces uncertainty about another (*Y*), reflecting potential co-occurrence or shared ecological function. CMI extends this by testing whether that association remains after accounting for the influence of other taxa (**Z**); in other words, it distinguishes direct associations driven by true biological dependencies from indirect ones mediated through other community members.

##### Overview of BNs

BNs are probabilistic graphical models that represent complex relationships among variables using directed acyclic graphs. Each node in a BN represents a random variable, while the directed edges capture conditional dependencies between them. BNs have been extensively applied in various biological network analyses, such as gene regulatory networks [24, 42, 43], but their use in microbiome research remains limited. There are three main approaches for learning the structure of BNs: Constraint-Based Methods: These are based on conditional independence tests to infer the network structure [23, 40, 42–47]. Score-Based Methods: These involve optimizing a scoring function to search among candidate network structures [48–50]. Hybrid Methods: These combine elements of both constraint-based and score-based approaches to leverage their respective strengths [22, 51–55]. Among these, the PC algorithm and its derivatives, such as Fast Causal Inference, Really Fast Causal Inference, and PCA-CMI [40, 46, 47, 56–58], are prominent constraint-based methods. Despite their widespread use, these methods have notable limitations: (1) Order Dependence: The results can vary depending on the sequence in which the nodes are processed [59]. (2) Static Threshold Dependency: Using fixed thresholds for conditional independence tests often leads to false positives or false negatives, reducing the reliability of inferred networks [42].

##### CMIMN algorithm

We propose the CMIMN algorithm to overcome the challenges posed by microbiome data, providing an order-independent, dynamic-threshold, and sparsity-controlled framework for microbiome network construction:

1. *Order independence:* Traditional PC-based methods, such as PCA-CMI, are susceptible to order dependence, where the sequence in which nodes are processed affects the inferred network structure. This occurs because, in these methods, the tests for conditional independence and edge removal are performed simultaneously during each iteration, making the results highly sensitive to the order of node traversal. In contrast, CMIMN eliminates this dependency by decoupling these steps. Specifically, for each step of the algorithm, CMIMN begins by fixing the set of potential separators for every edge (X,Y). This set is determined as the intersection of the neighbors of *X* and *Y* in the current graph. By defining the separators upfront, the algorithm ensures that all configurations are consistently evaluated, regardless of the order in which nodes or edges are processed. Once the potential separators are fixed, the algorithm proceeds to calculate the independence measures (e.g., CMI) for each edge using the predefined separator sets. Edges that fail the independence test, based on the dynamically determined thresholds, are then removed. This sequential separation of tasks (fixing separators, calculating independence measures, and then removing edges) ensures that the outcome of each step is independent of the traversal order of the nodes.
2. *Dynamic thresholds:* In traditional PC-based methods an edge between two nodes is removed if the independence measure (e.g., MI or CMI) falls below a predefined static threshold, usually 0.05. However, this fixed-threshold approach is inherently rigid and can lead to significant issues. Specifically, static thresholds are often poorly calibrated to the scale and variability of different datasets, resulting in false positives (retaining spurious edges) or false negatives (removing meaningful edges). To overcome these limitations, CMIMN employs a quantile-based dynamic threshold approach. All pair-wise MI values are first computed, and edges below the $q_{1}$ quantile are removed. This step retains a broad set of associations. Next, conditional MI is calculated for each remaining pair (X,Y) across all common neighbors **Z**, and the maximum CMI value is retained. Edges with maximum CMI below the $q_{2}$ quantile are removed, ensuring that only strong direct dependencies are preserved.
3. *Sparsity control:* The parameters $q_{1}$ and $q_{2}$ are user-defined and directly control network sparsity: lower quantiles yield denser graphs, while higher quantiles yield sparser, more conservative structures. In CMIMN, edge filtering is based on quantile thresholds rather than fixed cutoffs. Specifically, all pair-wise MI values are first computed, and edges below the 70th percentile are removed to retain a broad set of candidate associations. CMI is then calculated for the remaining pairs, and edges with maximum CMI values below the 95th percentile are discarded, ensuring that only strong direct dependencies are preserved. In this study, we set $q_{1}=70\%$ to maintain broad candidate associations at the MI stage and $q_{2}=95\%$ to focus on strong direct dependencies at the CMI stage. This choice balances sensitivity and specificity, producing networks that capture both broad and robust associations. If a gold standard network were available, $q_{1}$ and $q_{2}$ could be optimized using receiver operating characteristic-based analyses. Since no such ground truth exists in microbiome studies, we instead harmonized thresholds across methods so that the resulting networks had comparable edge densities, facilitating consensus rather than optimizing a single algorithm’s output.

The steps of the CMIMN algorithm are outlined below:

*Step 0: Initialization:* Generate a complete network with the number of nodes equal to the number of taxa.

*Step 1: Calculate MI of Order 0:* Compute MI values for each pair of nodes.

*Step 2: Remove edges:* Rather than using a fixed cutoff, we apply a quantile-based threshold. Specifically, all pair-wise MI values are computed, and edges with MI below the $q_{1}$ quantile (default $q_{1}=0.70$) are removed. This step retains the stronger 70% of associations, providing an initial filter that preserves broad relationships while discarding weak signals. The resulting network at this stage is denoted by $S_{0}$.

*Step 3: Calculate CMI of Order 1:* For each pair (X,Y) with an edge in $S_{0}$, calculate CMI(X,Y|Z) for all neighbors **Z** shared by *X* and *Y*. We retain the maximum CMI across all such **Z**, which reflects the strongest conditional dependency between *X* and *Y* after accounting for potential mediators.

*Step 4: Remove edges:* Edges are removed if their maximum CMI value is smaller than the $q_{2}$ quantile (default $q_{2}=0.95$) of the overall CMI distribution. This stricter threshold emphasizes strong direct dependencies while filtering out edges more likely explained by indirect effects. The resulting skeleton at this stage is denoted by $S_{1}$.

*Final outcome:* The resulting network $S_{1}$ is a fully undirected skeleton.

#### Normalization step in CMIMN

Normalization is an important preprocessing step before applying CMIMN to microbiome count data. Using raw counts without any transformation can lead to unstable MI and CMI estimates due to the large variability inherent in sequencing data. To evaluate the impact of normalization on CMIMN, we systematically compared four commonly used approaches: logarithmic transformation (Log), Centered Log-Ratio (CLR) [19], Geometric Mean of Pair-wise Ratios (GMPR) [60], and Total Sum Scaling (TSS). Each normalization method was applied to both the original dataset and 50 bootstrap datasets (see the Bootstrap robustness analysis section). The same set of bootstrap datasets was used for all normalization methods to ensure a fair comparison. For each normalization strategy, we computed F1-scores between the network inferred from the original data and the networks reconstructed from bootstrap datasets. To further assess how normalization affects the resulting network structure, we evaluated the similarity of networks obtained under different normalization methods. Networks were independently inferred from Log-, CLR-, GMPR-, and TSS-normalized datasets, and pair-wise F1-scores were computed between the constructed networks of every pair of normalization methods (e.g., CLR–GMPR, Log–CLR). This pair-wise comparison quantifies how normalization alone influences network structure. Based on these benchmarking analyses, Log transformation was selected as the default preprocessing step in CMIMN. Log transformation is widely recognized as a practical variance-stabilizing approach for count data [61] and has been shown to produce stable MI and CMI estimates across a range of datasets. However, because the performance of normalization strategies may vary across datasets [62], CMIMN allows users to externally apply alternative normalization methods such as CLR [19], GMPR [60], or TSS when appropriate. This flexible design ensures that researchers may select the preprocessing method most suitable for their data characteristics. CMIMN is implemented in R and publicly available at https://github.com/solislemuslab/CMIMN. When the quantitative argument is set to TRUE, input data are automatically log-transformed before MI and CMI estimation. While log transformation stabilizes variance, it does not fully resolve compositional dependencies; therefore, users retain full control to apply their preferred normalization strategy prior to running CMIMN. This modular framework ensures transparent and customizable data preprocessing without imposing a fixed normalization approach.

#### Constructing a consensus network

We apply four algorithms to construct microbiome networks. In each algorithm, the input is an abundance matrix, and the output is an undirected network in which nodes represent OTUs and edges correspond to interactions between them.

First, SPIEC-EASI [19] estimates sparse inverse covariance matrices to infer ecological associations in microbial communities. The approach is designed to address the challenges of compositional data and high dimensionality commonly encountered in microbiome studies. We employed the graphical lasso (glasso) variant of SPIEC-EASI (referred to as SE_glasso) rather than the Meinshausen–Bühlmann neighborhood selection variant. The goal of this study was to integrate distinct inference algorithms (CMIMN, SPARCC, SPRING, and SPIEC-EASI) into a consensus framework, rather than to benchmark SPIEC-EASI variants. The glasso approach has been widely used in soil microbiome studies and, based on bootstrap robustness analyses in our follow-up CMiNet package [25], showed more stable performance than Meinshausen–Bühlmann on the same dataset. For consistency and clarity, we therefore restricted our analysis to the glasso variant.

Second, the SPRING [20] is a statistical method designed to infer microbial association networks from quantitative microbiome data, which includes absolute microbial counts paired with sequencing data. To address the challenges posed by excess zeros and compositional effects, SPRING employs a semi-parametric rank-based estimator for correlations and partial correlations. This estimator is robust to data sparsity and sequencing depth limitations. By integrating this approach with sparse graphical modeling, SPRING enables reliable inference of ecological interactions among microbial taxa.

Third, SPARCC [18] is specifically designed for microbiome data represented as relative abundances. It addresses the constraints of nonnegative, compositional data by assuming a sparse underlying network and estimating correlations through an iterative algorithm. A bootstrap procedure is applied to assess significance and improve reliability. By revealing significant co-occurrence patterns and potential ecological interactions, SPARCC provides a valuable tool for investigating complex microbial ecosystems.

Fourth, CMIMN is a BN framework based on conditional MI that infers microbial interaction networks by capturing both linear and non-linear dependencies among taxa. Bootstrap resampling provides robustness assessment, and the algorithm has been shown to yield stable and interpretable ecological associations.

Building reliable microbiome networks is difficult because different algorithms produce varying results. Each method has its own strengths and weaknesses, which can lead to inconsistent interpretations of microbial interactions. To address this, we combined the results of the four algorithms (SE_glasso, SPRING, SPARCC, and CMIMN) into a consensus network. The consensus network is represented as a weighted adjacency matrix, where each edge is assigned a weight from 0 (not identified by any algorithm) to 4 (identified by all four algorithms), reflecting the level of agreement among methods. Among the advantages of the Weighted Consensus Network, we can highlight the *robustness* as integrating multiple algorithms reduces the impact of biases or errors associated with any single method; *stability* as the weighted approach provides a holistic view of microbial interactions, capturing edges that are consistently supported across algorithms; *interpretability* as the weight values offer a straightforward measure of edge confidence, allowing researchers to focus on highly reliable interactions for downstream analysis, and *sparsity Control* as selecting different threshold values for edge weights (e.g., retaining only edges with weights ≥1, ≥2, ≥3, or =4) can control the sparsity of the network to match their analytical goals. For instance, lower thresholds (e.g., ≥1) result in denser networks that include more potential interactions, while higher thresholds (e.g., =4) produce sparser networks focused only on the most reliable interactions identified by all algorithms. This weighted consensus serves as a stable foundation for exploring microbiome interactions and identifying key taxa.

We focused on the presence of strong associations rather than their sign. Accordingly, both positive and negative associations inferred by SPARCC (correlation-based) and by precision-matrix or rank-based methods such as SE_glasso and SPRING were treated equally when integrating into the consensus network. Because MI- and CMI-based methods (e.g., CMIMN) return unsigned dependencies, we harmonized all methods by considering only the strength of associations, without distinguishing directionality. Thus, the consensus edges in this study should be interpreted as unsigned dependencies, representing reproducible connections without distinguishing between cooperative and competitive interactions.

The consensus network in this study was constructed using a simple voting strategy, where edge weights reflect the number of methods supporting a given association. This approach was chosen to provide a transparent and computationally efficient way of capturing agreement across inference methods. This design allowed us to first identify broadly supported associations and then evaluate their robustness under resampling, ensuring both interpretability and reliability. We note that our subsequent CMiNet package [25] builds on this framework by integrating additional inference methods and incorporating bootstrap-based reproducibility analysis directly into consensus construction.

### Evaluation design

#### Bootstrap robustness analysis

To assess the reproducibility of the CMIMN algorithm in learning the microbiome network, we conducted a comprehensive bootstrap analysis. In the first step, we constructed the network using all available samples. We then evaluated robustness by generating 50 bootstrap replicates, where each dataset was created by randomly resampling with replacement from the full set of samples, maintaining the original dataset size. Each bootstrap dataset was analyzed independently with the given algorithm to construct separate networks. For each method, we compared the networks constructed from the 50 generated datasets to the corresponding network built using the original dataset. Two complementary measures were used: (1) the F1-score, which balances precision and recall to quantify agreement between bootstrap-derived networks and the reference network, and (2) the Jaccard similarity index [63], which measures the proportion of shared edges relative to the union of edges in the two networks. Both metrics evaluate reproducibility under resampling rather than biological accuracy. Higher scores indicate greater stability of inferred associations.

#### Synthetic study

To further assess the performance of CMIMN beyond robustness analyses on experimental data, we evaluated its ability to recover known associations using synthetic data with a defined reference network. Because true microbial interaction networks are unknown in real datasets, synthetic datasets with known ground-truth structures enable direct benchmarking of algorithmic performance [19, 20]. We therefore generated synthetic microbiome data with the SPRING package [20], which simulates microbial count data from a prescribed band graph structure [19]. This design allows direct comparison of each inferred network against the known ground-truth correlation matrix. The synthetic dataset was based on the American Gut Project (127 taxa, 289 samples) [19]. We applied four inference methods, including SE_glasso [19], SPRING [20], SPARCC [18], and our proposed CMIMN, and compared the resulting networks with the true adjacency matrix. To ensure fairness, the parameters of each method were tuned so that the number of inferred edges was comparable to the ground-truth network. Performance was evaluated using standard edge-recovery metrics, including precision, recall, and F1-score.

### Multi-method approach to identify key microbial drivers of disease resistance

Feature selection is a crucial step in data analysis, involving the identification of significant features or covariates that possess high predictive power. In the context of high-dimensional data, such as microbiome datasets, feature selection becomes indispensable to extract relevant information and reduce computational complexity. In particular, when studying diseases, it becomes imperative to identify important OTUs that are strongly associated with the disease’s onset or progression. By identifying these key OTUs, we gain essential insights into potential driver pathogens or beneficial microbes. Subsequently, controlling the abundance or activity of these crucial OTUs can pave the way for novel disease interventions and management strategies, opening up avenues for precision medicine and tailored therapies. To identify key OTUs associated with disease outcomes, we employ a two-pronged feature selection approach: (1) ML-based methods, and (2) Network-based methods.

#### ML-based methods

To identify important OTUs, we first normalize the filtered microbiome data using different transformations: CLR, which transforms relative abundances by log-ratio scaling to remove compositional effects and make the data more suitable for standard statistical models, raw filtered data, where no further transformation is applied beyond initial filtering, serving as a baseline representation, logarithmic transformation (log), which reduces the influence of highly abundant taxa, and TSS, which normalizes each sample by dividing feature counts by the total counts per sample, converting counts into relative abundances. We then apply all ML-based feature selection strategies, implemented in the scikit-learn library [64] in Python: (1) “SelectKBest” method, which ranks features by univariate statistical tests and selects the *k* highest-scoring features. Specifically, we used the analysis of variance F-test and MI for classification, both of which assess the dependence between each feature and the binary disease outcome, (2) Selection of the top *k* features based on the MI statistic (a non-parametric score that estimates both linear and non-linear dependencies between individual features and the target), (3) Recursive Feature Elimination (RFE) with logistic regression (an iterative method that fits a logistic regression model, ranks feature importance by model coefficients, and removes the least important features step-by-step), (4) RFE with a decision tree (similar iterative elimination process, but using decision tree feature importance scores to guide selection, thus capturing nonlinear interactions), (5) RFE with gradient boosting (leveraging boosted decision trees that sequentially correct errors of prior models, providing a strong non-linear ranking of features), and (6) RFE with Random Forest (RF) (using an ensemble of decision trees with bootstrapped sampling and random feature splits, which provides stable and robust measures of variable importance). Additionally, we introduce a seventh method that includes OTUs in the model if their maximum abundance falls within the top 20% of the dataset (a threshold-based filter designed to retain highly abundant taxa that may be overlooked by model-driven selection). For all methods, we selected only the top 20% of OTUs as important, ensuring that only the most strongly prioritized features were retained. For more details on feature selection methods, see [2].

To aggregate the results across all seven ML-based strategies, we assigned a TOTAL score to each OTU, representing the number of methods in which the OTU was selected as important. For example, an OTU selected by all seven methods receives a TOTAL score of 7, while one selected by only three methods receives a score of 3. This score quantifies cross-method consensus and highlights taxa consistently prioritized across diverse modeling approaches. Because only the top 20% of features from each method contribute to the TOTAL score, the design helps mitigate concerns that moderately ranked taxa might be overweighted across models. This conservative strategy increases confidence that high TOTAL scores correspond to truly influential OTUs rather than artifacts of averaging. In practice, we used a majority-rule cutoff (TOTAL > 3) as the default threshold, retaining only taxa supported by more than half of the ML methods. This balances inclusiveness and rigor by prioritizing reproducible signals while avoiding overly stringent cutoffs. As an alternative, one could also apply a quantile-based threshold (e.g., top 20% of TOTAL scores), analogous to the network-based feature selection framework [25].

#### Network-based methods

While ML-based methods identify statistically relevant OTUs, they do not capture microbial interactions that may play a crucial role in disease resistance. To address this limitation, we employ a network-based feature selection strategy that compares microbial interaction networks between diseased and healthy samples. We construct microbial interaction networks using four well-established methods (1- SPARCC, 2- SE_glasso, 3- SPRING, and 4- CMIMN) based on samples from two classes: one representing samples without the disease (‘clean tubers’) and the other with the disease (“scab-infected tubers”). We then apply two complementary network-based feature selection strategies:

##### Strategy 1: differential centrality analysis

This approach analyzes five centrality metrics for each OTU: (1) Degree: number of direct connections, highlighting hub taxa with many associations. (2) Eigenvector centrality: importance based on being connected to other influential taxa, identifying globally influential members. (3) PageRank (PR): importance derived from the link structure, highlighting taxa that remain central even in sparse or hierarchical networks. (4) Closeness: overall proximity to all other taxa, reflecting how quickly a taxon can influence or be influenced by the network. (5) Betweenness: frequency of lying on shortest paths, indicating taxa that act as bridges linking different parts of the community. We rank OTUs based on the difference in their centrality measures between the “clean tubers” and “scab-infected tubers” networks. The top 20% of OTUs showing the most significant variations are selected as key taxa. The final taxa are considered important if they are selected by all four network inference methods.

##### Strategy 2: weighted scoring of OTUs based on network topology

This method assigns a weighted score to each OTU based on its network properties using the following formula:


(5) $$\mathrm{Scor}e_{i}^{j}=w_{1}\times {\text{DE}}_{i}^{j}+w_{2}\times {\text{EV}}_{i}^{j}+w_{3}\times {\text{PR}}_{i}^{j}+w_{4}\times {\text{CL}}_{i}^{j}+w_{5}\times {\text{BE}}_{i}^{j}$$

where, *i* denotes the OTU being evaluated and *j* represents the network inference method used for constructing the microbiome network. The weights assigned to the centrality measures reflect their relative importance in capturing biologically meaningful insights from the network structure. To ensure that the OTUs identified as significant have greater overlap with Strategy 1, we set the weights as follows: $w_{1}=0.1,w_{2}=0.1,w_{3}=0.1,w_{4}=0.2,w_{5}=0.5$. We placed the highest weight on betweenness centrality ($w_{5}=0.5$) because prior studies have shown that taxa with strong bridging roles, often captured by betweenness, are critical for identifying keystone species and maintaining community structure [12, 65]. Closeness centrality was assigned a moderate weight ($w_{4}=0.2$) because it captures global influence efficiency: how quickly a taxon can interact with or be affected by others across the entire network, reflecting its capacity to spread effects or respond rapidly within the community [65–67]. In contrast, degree, eigenvector, and PageRank were each assigned lower weights (w=0.1), as they primarily describe local connectivity or influence through immediate neighbors rather than cross-community control points. Grouping these measures into two families-path-based (betweenness, closeness) and neighbor-based (degree, eigenvector, PageRank), we allocated a higher total weight to the path-based family (0.7) because these metrics capture both global communication efficiency and structural cohesion. These rounded values (0.5, 0.2, 0.1) were chosen for clarity and reproducibility; exploratory tests of alternative weightings produced consistent overlap with Strategy 1 (differential centrality analysis). A full sensitivity analysis was beyond the scope of this study but represents an important direction for future work.

The final top 20% of OTUs with the highest scores are considered key players in microbial interactions related to disease resistance. We selected the top 20% of OTUs in both network-based strategies. This threshold was chosen as a pragmatic compromise: stricter cutoffs (e.g., top 10%) reduced the overlap between ML-based and network-based strategies, whereas more permissive cutoffs (e.g., top 30%) tended to include weaker signals. Thus, the 20% cutoff provided a balanced choice that maximized reproducibility across strategies while retaining interpretability. We note that in general, thresholds between 10%–30% can highlight important taxa, but here we opted for 20% to ensure a robust intersection across methods. Importantly, $q_{\text{net}}$ is a user-defined parameter in our framework and can be adjusted (e.g., 10%–30%) depending on study goals.

##### Unifying machine learning and network-based approaches for reliable microbiome feature selection

To improve the reliability of microbiome feature selection, we integrate both ML and network-based strategies. Specifically, we evaluate OTUs identified by two different network-based methods with those selected through multiple ML algorithms. The final set of selected OTUs consists of taxa consistently prioritized across these complementary approaches, thereby increasing confidence in their biological relevance. This integrative strategy combines the predictive power of ML with the structural insights derived from microbial interaction networks, resulting in a robust and interpretable set of microbial features. The selected OTUs represent strong candidates for microbial drivers of disease resistance and may inform the development of microbiome-targeted interventions aimed at enhancing crop resilience and promoting sustainable agricultural practices.

##### Workflow overview

**Figure 1.**
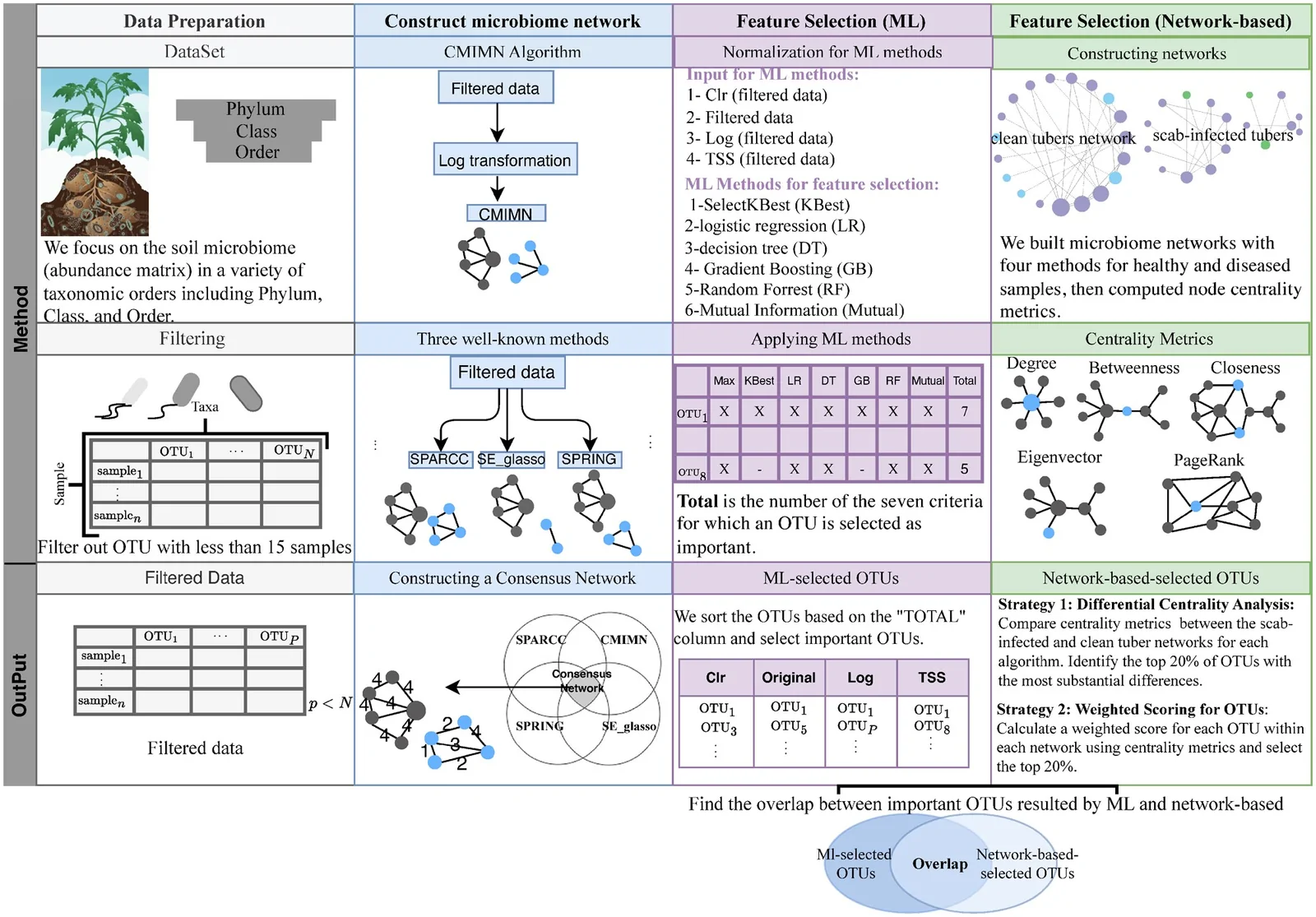
Workflow of the microbiome analysis pipeline for identifying key microbial drivers of disease resistance. Using potato common scab as an example, this pipeline consists of five main steps and is generally applicable to any microbiome dataset: Data Preparation: Raw microbiome data is preprocessed to retain OTUs present in at least 15 samples, ensuring that low-prevalence taxa do not introduce noise. The resulting filtered data matrix serves as the foundation for downstream analysis, focusing on taxonomic levels such as Phylum, Class, and Order. Construct microbiome network: Microbiome networks are constructed using four inference methods: SE_glasso, SPRING, SPARCC, and the proposed CMIMN. These networks represent microbial taxa as nodes and their interactions as edges. To improve reliability, a consensus microbiome network is constructed by integrating results from all four methods. Edge weights indicate the level of agreement among methods, with a weight of 4 representing relationships confirmed by all methods and 0 indicating no agreement. Feature selection (ML): The filtered data undergoes normalization using three methods (CLR, log, and TSS) before applying ML-based feature selection methods. A TOTAL score is assigned to each OTU based on its selection frequency across ML methods, identifying key taxa strongly associated with disease outcomes. Feature selection (network-based): Microbiome networks are separately constructed for “scab-infected” and “clean tuber” samples. Two strategies are applied to identify key OTUs based on network structure: (1) Differential Centrality Analysis, and (2) Weighted scoring of OTUs. Final OTU selection (identifying overlap): The last step identifies the overlap between OTUs selected by ML-based and network-based approaches, ensuring robust and reliable feature selection for downstream microbiome analysis

Figure 1 provides a visual summary of the full analysis pipeline described in this section, from data preparation through network construction, feature selection (ML and network-based), and the final identification of important taxa using multiple strategies. For exact settings (e.g., prevalence filters, normalization options, network thresholds, and selection cutoffs) and software used, see Table S4.

## Results

### Effect of normalization on CMIMN stability

Analyses were performed on the American Gut dataset (amgut1 from the SpiecEasi package [19], containing 289 samples and 127 OTUs) and on soil microbiome datasets aggregated at the Phylum, Class, and Order levels. Figure S1 (see online supplementary material for a colour version of this figure) shows the distribution of F1-scores obtained by comparing the network inferred from the original dataset with networks reconstructed from the 50 bootstrap datasets across all datasets (amgut1, Soil_Phylum, Soil_Class, Soil_Order). Each boxplot represents the range of 50 F1-scores for one normalization method. Across all datasets, normalization had a measurable effect on network reproducibility. For the American Gut dataset, Log transformation achieved the highest median F1-score (0.75) and the lowest standard deviation (SD) (0.02). At the Class level, all normalization methods performed similarly. At the Phylum and Order levels, Log transformation again produced higher median F1-scores and lower variability compared to CLR, GMPR, and TSS (Table S1). In this table, the first column lists the datasets, the second column indicates the normalization method, the third column reports the median of the 50 F1-scores, and the fourth column provides the corresponding SD. Table S2 summarizes the pair-wise comparisons between networks inferred under different normalization methods. For each pair of methods (e.g., CLR–GMPR), networks were constructed from the 50 bootstrap datasets using each normalization method, resulting in two sets of 50 networks. The F1-score was then computed for each pair of networks generated from the same bootstrap dataset, yielding 50 F1-scores per normalization pair. The median and SD of these values are reported in Table S2. Networks inferred from Log-normalized data showed moderate to high overlap with those inferred from CLR-, GMPR-, and TSS-normalized data. GMPR–TSS pairs consistently exhibited the highest structural similarity across datasets, while pairs involving CLR (e.g., CLR–GMPR, CLR–TSS) showed the lowest overlap. These results demonstrate that different normalization strategies can lead to differences in the inferred network structure.

### Robustness study of different algorithms for learning the microbiome network

We visualized the stability of each method’s performance using box plots of the F1-score and Jaccard distributions across taxonomic levels (Phylum, Class, and Order) (Fig. S2, see online supplementary material for a colour version of this figure). These plots revealed that CMIMN consistently displayed relatively narrow distribution ranges across all taxonomic levels, suggesting stable reproducibility. In contrast, SPRING showed the least favorable performance. To further quantify robustness, we calculated the mean F1-score, SDs, and 95% bootstrap percentile confidence intervals (CIs) from 50 replicates for each algorithm and taxonomic level (Table S5). The CI was computed using the percentile method [68], defined as $\left(\theta_{0.025}^{*},\theta_{0.975}^{*}\right)$, where $\theta^{*}$ denotes the bootstrap estimate of the F1-score. Algorithms with narrow CIs and small SDs demonstrate stable performance, whereas wider intervals indicate greater sensitivity to variation in the input data. Across taxonomic levels, SPRING and CMIMN generally had the narrowest CIs, indicating more stable reproducibility, whereas SE_glasso showed the widest intervals, reflecting greater variability.

In addition, we performed pair-wise statistical comparisons between algorithms using the Wilcoxon signed-rank test on the bootstrap-derived F1-scores. Pair-wise heatmaps (Fig. S3, see online supplementary material for a colour version of this figure) illustrate median differences (Method A minus Method B) along with significance levels, where arrows and stars denote the direction and strength of the difference. Complete pair-wise statistics, including median F1-scores and adjusted Wilcoxon *P*-values for all method comparisons, are summarized in Table S6. Overall, CMIMN achieved the highest and most consistent F1-scores across all taxonomic levels, significantly outperforming other methods in most comparisons. In contrast, SPARCC and SE_glasso exhibited relatively similar performance patterns, whereas SPRING differed substantially from the others, showing notably lower agreement with empirical data.

### Validation on synthetic data

To further assess the performance of CMIMN beyond robustness analyses on experimental data, we evaluated its recovery of known interactions on synthetic data with a defined reference network. Among the tested methods, SPRING achieved the highest overall F1-score (0.94). SE_glasso and CMIMN followed closely with F1-scores of 0.79 and 0.77, respectively, while SPARCC yielded lower performance (F1-score = 0.70). These results demonstrate that CMIMN performs competitively against established inference methods, and full results are provided in Table S7.

### Minimal overlap across network inference methods highlights the need for using a consensus approach

We constructed microbiome networks using four different inference methods: (SE_glasso, SPRING, SPARCC, and CMIMN) at the Phylum, Class, and Order levels. Despite using the same dataset, the resulting networks exhibited minimal overlap, highlighting the high variability in microbial interaction patterns inferred by different methods. To assess computational efficiency, we benchmarked run-times of all methods at the Phylum, Class, and Order levels (38, 99, and 189 taxa; n=214 samples). CMIMN completed in 0.139-9.052 s, substantially faster than SE_glasso (8.276-177.559 s; ≈3.0 min at 189 taxa) and SPRING (28.681-862.286 s; ≈14.4 min at 189 taxa), and comparable to SPARCC (0.205-4.211 s; see Table S8). Run-time scaled with the number of taxa, but all methods completed comfortably on a workstation using eight CPU cores and 16 GB of RAM. These results suggest that CMIMN is computationally efficient and scalable for larger microbiome datasets.

**Figure 2.**
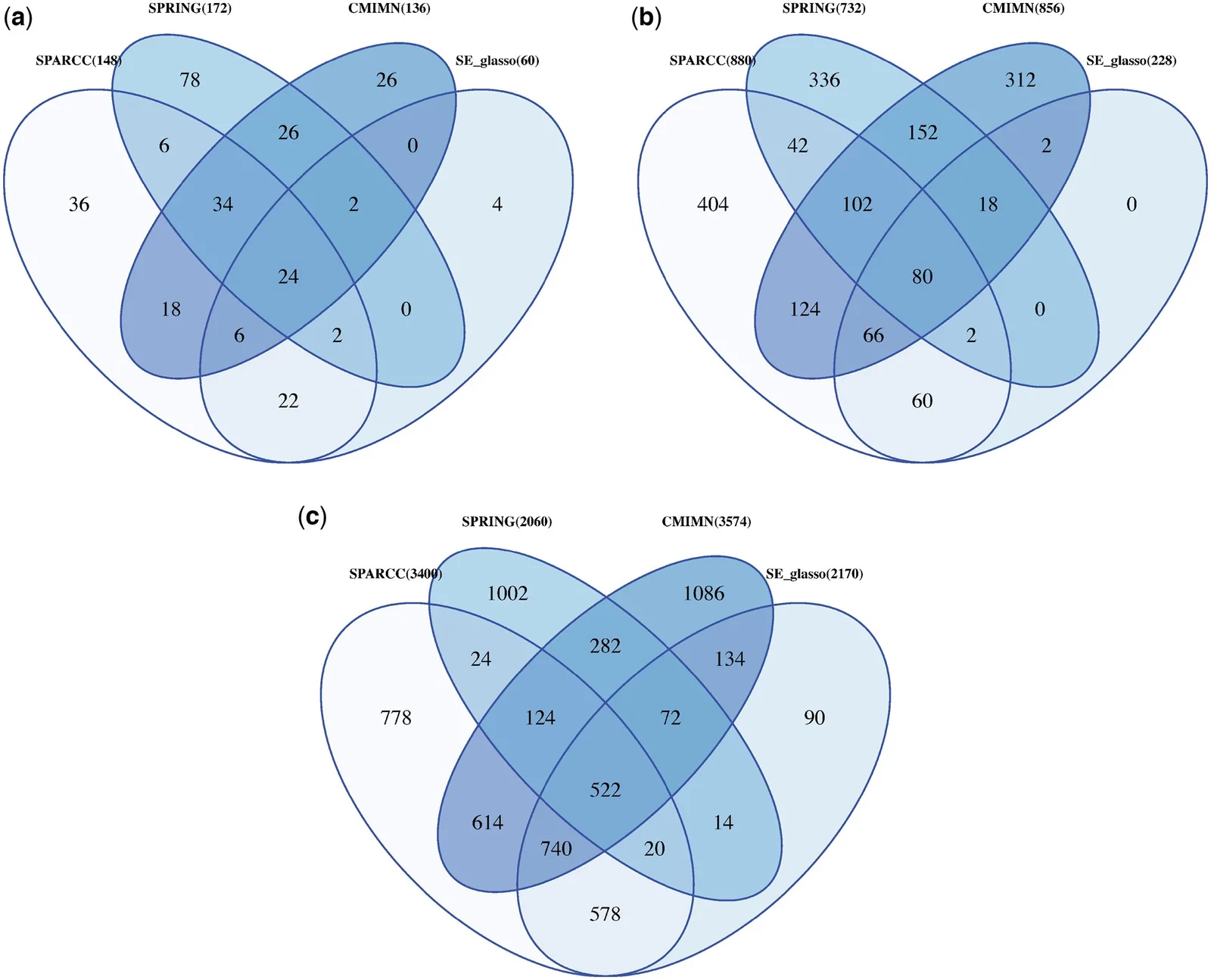
Venn diagrams illustrating the overlap of common edges in microbiome networks constructed using four different inference methods (SE_glasso, SPRING, SPARCC, and CMIMN) at different taxonomic levels based on all samples. (**a**) Phylum level: 24 common edges among all methods. (**b**) Class level: 80 common edges. (**c**) Order level: 522 common edges

Figure 2 shows the Venn diagrams of common edges inferred by different methods at different taxonomic levels: only 24 common edges at the Phylum level, 80 at the Class level, and 522 at the Order level. These results indicate that network structures can vary significantly depending on the inference method used, which raises concerns about the reliability of conclusions drawn from any single approach.

Tables S9, S10, and S11 present network metrics for the four different methods at the Phylum, Class, and Order levels. The substantial differences in network topology metrics underscore the inherent differences in algorithmic assumptions and their impact on inferred microbial interactions. This variation highlights the importance of employing a consensus-based approach to enhance network robustness, reduce algorithm-specific biases, and improve biological interpretability.

Tables S12, S13, and S14 present the top nodes in microbial networks based on topological measures at the Phylum, Class, and Order levels. These networks were constructed using SE_glasso, SPRING, SPARCC, and CMIMN methods, and the analysis encompasses data from all samples, without distinguishing between diseased and healthy conditions.

Interestingly, certain OTUs were consistently identified as highly connected nodes across all four network construction methods, reinforcing their biological significance. According to these tables, *Acidobacteriae* was identified as important at the Class level by all algorithms and network metrics, while *C0119* was consistently identified at the Order level. The recurrence of these taxa across multiple network inference methods suggests their potential ecological importance and role in microbial community stability.

### Consensus microbiome network: enhancing reliability through integration

**Figure 3.**
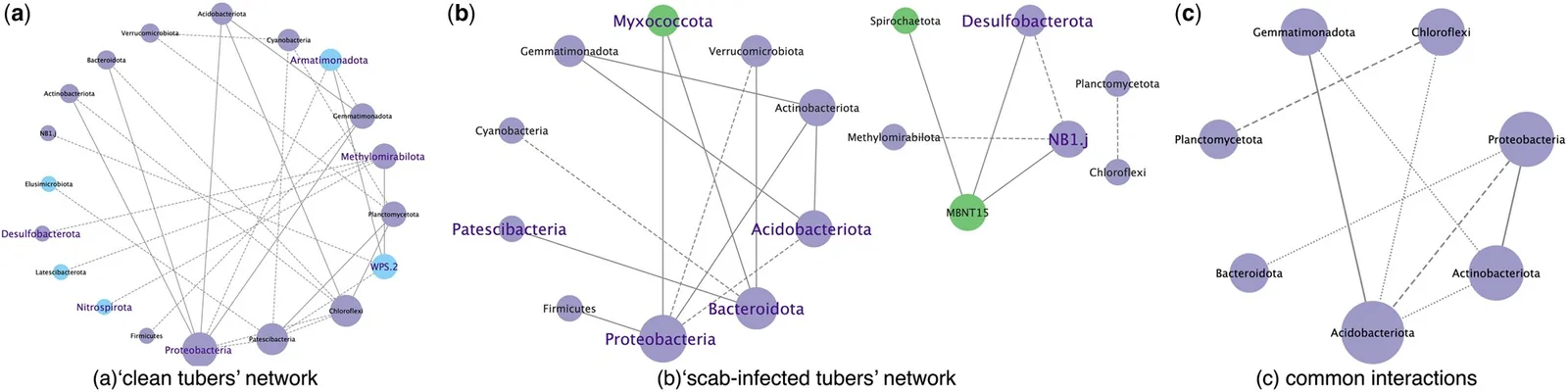
The microbiome network at the Phylum taxonomic level. Part (**a**) represents the “clean tubers” network, part (**b**) displays the “scab-infected tubers” network, and part (**c**) shows the common interactions between them. Nodes represent OTUs and are color-coded: purple for common OTUs shared between “clean tubers” and “scab-infected tubers” networks, blue for OTUs unique to the “clean tubers” network, and green for OTUs unique to the “scab-infected tubers” network. Node size indicates their degree of connectivity. Edges are categorized as dashed lines (confirmed by three methods) or solid lines (confirmed by all four methods)

Figures 3 and Figs S4 and S5 (see online supplementary material for a colour version of these figures) visualize the microbiome networks at the Phylum, Class, and Order levels respectively. Part (a) of each figure represents the “clean tubers” network, which is characterized by a denser and more connected microbial community, with more diverse interactions. Part (b) shows the “scab-infected tubers” network, highlighting nodes and edges unique to diseased samples. Part (c) illustrates the common interactions between the two networks. Nodes represent OTUs and are color-coded: Purple: Common OTUs shared between “clean tubers” and “scab-infected tubers” networks. Blue: OTUs unique to the “clean tubers” network. Green: OTUs unique to the “scab-infected tubers” network. Node size reflects connectivity (degree), while edges are distinguished as dashed (confirmed by three methods) or solid (confirmed by all four methods). At the Class and Order levels, due to the density of edges, only solid edges (confirmed by all four methods) are reported for clarity.

In both “clean tubers” and “scab-infected tubers” microbiome networks, we identified microbial associations consistently supported by all four network inference methods. Many of these associations have also been independently reported as ecologically meaningful in soil ecosystems. The intersections of microbial associations between the clean tubers’ and scab-infected tubers’ networks are summarized in Tables S15, S16, and S17, corresponding to the Phylum, Class, and Order levels, respectively. Each table includes three columns: the first lists associations unique to the “clean tubers” network, the second shows associations exclusive to the “scab-infected tubers” network, and the third highlights shared associations, those present in both networks, representing conserved or stable microbial interactions across conditions.

Due to the large number of associations observed at the Class and Order levels, we report the complete set of edges confirmed by all four methods in Supplementary Tables S16 and S17. Here, we focus on selected Phylum-level associations that are most frequently supported by existing literature. In the Table S15 for “clean tubers” network, we observed the interaction between *Planctomycetota–Patescibacteria*, which likely reflects syntrophic or symbiotic interactions, as both phyla possess reduced genomes and are known to co-occur in structured soil aggregates [69]. The edge between *Methylomirabilota–WPS-2* may indicate shared adaptation to oligotrophic or co-contaminated soil conditions, where both phyla are often involved in carbon and nitrogen cycling under stress [70]. Additionally, *Gemmatimonadota–Proteobacteria* and *Gemmatimonadota–Acidobacteriota* were found only in the clean network; these phyla are often associated with nutrient cycling and stable soil conditions, suggesting cooperative metabolic roles in healthy tuber-associated soils.

In the “scab-infected” network, the *Actinobacteriota–Gemmatimonadota* interaction was unique and mirrors findings from boreal forest soils, where both phyla jointly contributed to the transformation of dissolved organic matter during freeze–thaw cycles, pointing to potential functional synergy under stress [71].

In both “clean tubers” and “scab-infected” networks, the edges *Actinobacteriota–Proteobacteria* and *Proteobacteria–Acidobacteriota* were consistently present, suggesting stable and ecologically relevant relationships across conditions. The association between *Actinobacteriota and Proteobacteria* has been reported in sandy and layered soils, where both phyla are dominant and likely contribute to complementary roles in organic matter degradation and nutrient cycling [72]. Meanwhile, *Proteobacteria* and *Acidobacteriota* are among the most abundant phyla in forest and agricultural soils and are known to occupy distinct but co-existing niches, with *Proteobacteria* favoring copiotrophic (nutrient-rich) environments and *Acidobacteriota* preferring oligotrophic, acidic soils, indicating a functional partitioning that supports broad microbial diversity and resilience [73].

### Feature selection using a multi-method approach

#### Machine learning-based feature selection

Tables S18, S19, and S20 summarize the results of selected features based on ML methods at the Phylum, Class, and Order levels, respectively. Each table includes five columns: four corresponding to different data normalization strategies applied prior to ML analysis, and a fifth representing their intersection. The first column (ML_CLR) reports OTUs selected from count-filtered data normalized using the CLR transformation. The second column (ML_Original) shows OTUs selected from raw count-filtered data without transformation. The third column (ML_Log) includes OTUs identified from log-transformed data, and the fourth column (ML_TSS) presents selections based on data normalized using TSS. The final column (ML_Intersection) lists OTUs consistently identified as important across all four normalization methods, highlighting microbial taxa that are robust to normalization choice. According to these tables, there is a good overlap between different normalization methods.

#### Network-based selection: strategy 1 (differential centrality analysis)

Tables S21, S22, and S23 provide lists of selected OTUs at the Phylum, Class, and Order levels, respectively, based on Strategy 1: Differential Centrality Analysis. OTUs were selected according to two criteria: (1) those exhibiting the largest differences in centrality values between “clean tubers” and “scab-infected tubers” networks, and (2) those consistently identified across all four network inference methods (SE_glasso, SPRING, SPARCC, and CMIMN), reinforcing their biological relevance. The first column of these tables shows the OTU name, while the second column (Features) indicates the centrality measures responsible for the OTU’s selection. These taxa represent microbial features whose connectivity patterns consistently differ between ‘clean tubers’ and ‘scab-infected tubers’ networks, suggesting a potential role in disease dynamics.

#### Network-based selection: strategy 2 (composite scoring approach)

First, we constructed two distinct microbiome networks: one for “clean tubers” and one for “scab-infected tubers.” Networks were generated using four inference methods: SE_glasso, SPRING, SPARCC, and CMIMN.

Next, we applied a composite scoring system that integrates multiple centrality metrics into a single weighted score for each OTU, as defined in Equation (5). This score was calculated separately for each of the four network inference methods. The top 20% of OTUs with the highest scores were considered significant. To evaluate the agreement between network-based selection (Strategy 2) and ML-based selection, we examined the overlap between the top 20% highest-scoring OTUs and those selected by ML-based methods.

Tables S24, S25, and S26 summarize these overlaps at the Phylum, Class, and Order levels, respectively. In these tables, the left section corresponds to results from the “clean tubers” network, while the right section presents the results from the “scab-infected tubers” network. Each section contains five columns: The first column lists the OTUs selected by Strategy 2. Columns 2–5 indicate whether the same OTUs were also identified by ML methods under four normalization strategies: CLR transformation, untransformed (raw) data, log-transformation, and TSS normalization. A value of “1” in these columns denotes agreement between Strategy 2 and the corresponding ML method for that normalization, while “0” indicates the OTU was uniquely selected by the network-based approach. This design enables a direct comparison of method overlap under different data preprocessing conditions. Figures S6 and S7 (see online supplementary material for a colour version of these figures) provide a visual representation of these overlaps, illustrating the average agreement between ML-based and network-based selection methods under different conditions. For “clean tubers,” at the Phylum level, CMIMN and SPARCC demonstrated slightly better agreement with ML-based selection across different normalization strategies. At the Order level, agreement was highest overall, particularly under CLR and TSS normalization. For “scab-infected” tubers, CMIMN generally exhibited higher agreement with ML-based selection at the Phylum level, while SPARCC showed better alignment at the Class level in certain cases. At the Order level, both CMIMN and SPARCC consistently achieved strong agreement with ML-based methods. The dashed horizontal lines in Figs S6 and S7 (see online supplementary material for a colour version of these figures) indicate the overall mean agreement across all methods and normalization strategies, providing a baseline for comparison. Points above the dashed line therefore represent above-average concordance between ML-based and network-based selections, while points below it indicate weaker-than-average alignment. These findings indicate that CMIMN and SPARCC consistently align more closely with ML-based feature selection methods, particularly at the Order level and under CLR and TSS normalization. This underscores their robustness and reliability in identifying important OTUs across different experimental conditions.

Tables S27, S28, and S29 present the overlap between Strategy 2 and the ML approaches at the Phylum, Class, and Order levels, focusing specifically on OTUs that were commonly identified in both the “clean tubers” and “scab-infected tubers” networks for each method. The left sections of these tables report overlaps for the CMIMN and SE_glasso methods. The first column lists the microbial taxa consistently identified across both networks using each respective method. Columns 2 through 5 indicate whether these taxa were also selected by ML methods under different normalization strategies. A value of “1” denotes agreement between the ML and network-based methods, while “0” indicates no overlap. Similarly, the right sections of the tables summarize the results for the SPARCC and SPRING methods, following the same structure.

Finally, Tables S30 and S31 illustrates the overlap among important OTUs identified by all four algorithms (CMIMN, SPARCC, SE_glasso, SPRING) for the “clean tubers” and “scab-infected tubers” networks, respectively, based on Strategy 2.

### Overall OTUs identified by all methods as key drivers of disease

To determine appropriate thresholds for identifying significant taxa, we systematically evaluated how the number and consistency of selected OTUs changed across a range of cutoff values in both ML-based and network-based frameworks (Figs S8 and S9, see online supplementary material for a colour version of these figures). For ML-based feature selection, we used the TOTAL score, which quantifies the number of ML algorithms (out of seven) in which each OTU was selected as important. By examining different cutoffs (TOTAL > 2, > 3, > 4, > 5) across four normalization strategies (CLR, raw, log, and TSS), we observed that a threshold of TOTAL > 3, meaning that a taxon must be supported by at least four ML algorithms, produced a reasonable number of taxa. At this level, 4, 11, and 25 taxa were identified at the Phylum, Class, and Order levels, respectively, all of which were consistently supported by multiple ML algorithms and normalization methods (listed in Table 1). For network-based selection (Strategy 2), we compared thresholds corresponding to the top 10%, 20%, and 30% of taxa ranked by their composite centrality scores. These scores integrate five network topology metrics (Degree, Eigenvector, PageRank, Closeness, and Betweenness) across four network inference methods (CMIMN, SPARCC, SPRING, and SE_glasso) and two network conditions (healthy and diseased). Figure S9 (see online supplementary material for a colour version of this figure) illustrates how the number of significant taxa changes with each selection threshold (10%, 20%, 30%) at different taxonomic levels (Phylum, Class, and Order). Each panel shows four bars representing: (1) the total number of taxa identified within each network (gray), (2) the number of OTUs consistently selected in the healthy (clean tubers) network by all four methods (red), (3) the number of OTUs consistently selected in the diseased (scab-infected tubers) network by all four methods (green), and (iv) the union of taxa from the healthy and diseased networks (purple). The top 20% cutoff was ultimately selected because it provided a reasonable number of taxa; stricter thresholds (10%) yielded too few overlapping taxa between healthy and diseased networks, whereas more lenient thresholds (30%) introduced low-confidence nodes with weak centrality support. In both network-based strategies, we set the selection cutoff at the top 20% of taxa, ensuring consistency between Strategy 1 (differential centrality analysis) and Strategy 2 (composite scoring approach) when identifying significant taxa. Importantly, these thresholds are not hyper-parameters of the algorithms but rather empirical criteria used to define the subset of top-ranked taxa for comparative visualization and biological interpretation. We adopted a less stringent threshold in the ML framework to capture a broader set of candidate taxa and a more stringent threshold in the network-based framework to isolate the most robust and reliable taxa. This flexible approach allows researchers to tailor the threshold selection to the goals and data characteristics of each study. The final taxa obtained using these thresholds (summarized in Table 1) represent a small yet highly reliable set of candidates consistently supported by both ML and network-based selection frameworks.

**Table 1.** Important taxa at the Phylum, Class, and Order levels across three different strategies.

Level	ML	Strategy 1	Strategy 2	Intersection
**Phylum**	*Firmicutes*	*Bacteroidota*	*Bacteroidota*	*Bacteroidota*^d^
	*Cyanobacteria*	*WPS-2*	*WPS-2*	*WPS-2*^d^
	*Armatimonadota*	*Proteobacteria*	*Proteobacteria*	*Proteobacteria*^d^
	*NB1-j*			
**Class**	*Bacilli*	*Desulfotobacteriia*	*Ktedonobacteria*	*Actinobacteria*^d^
	*Ktedonobacteria*	*Actinobacteria*	*Vicinamibacteria*	*AD3*^d^
	*Cyanobacterii*	*Syntrophobacteria*	*Actinobacteria*	*Bacilli*^c^
	*Saccharimonadia*	*AD3*	*Gammaproteobacteria*	*Anaerolineae*^c^
	*Planctomycetes*		*Alphaproteobacteria*	*Ktedonobacteria*^c^
	*Ignavibacteria*		*Acidobacteriae*	
	*Dehalococcoidia*		*Anaerolineae*	
	*Anaerolineae*		*AD3*	
	*MB.A2.108*		*Blastocatellia*	
	*Chthononomadetes*		*Bacilli*	
	*Kryptonia*			
**Order**	*Saccharimonadales*	*C0119*	*C0119*	*C0119* ^a^
	*Bacillales*	*Defluviicoccales*	*Rhizobiales*	*Defluviicoccales*^b^
	C0119	*Bacteroidales*	*Chitinophagales*	*Bacteroidales*^b^
	*Subgroup.2*	*Kryptoniales*	*Ktedonobacterales*	*Ktedonobacterales*^c^
	*Xanthomonadales*	*B12.WMSP1*	*Microtrichales*	
	*Acidobacteriales*	*Desulfotobacteriales*		
	*Chloroplast*			
	*Alicyclobacillales*			
	*Paenibacillales*			
	*Acetobacterales*			
	*Pseudomonadales*			
	*Anaerolineales*			
	*Elsterales*			
	*Bacteroidales*			
	*Ktedonobacterales*			
	*Sphingomonadales*			
	*Kineosporiales*			
	*SBR1031*			
	*Rokubacteriales*			
	*Frankiales*			
	*Micropepsales*			
	*Gaiellales*			
	*PLTA13*			
	*Defluviicoccales*			
	*Obscuribacterales*			

The superscript alphabets indicate intersections between the levels:

a For taxa appearing in ML, Strategy 1, and Strategy 2.

b For taxa appearing in ML and Strategy 1.

c For taxa appearing in ML and Strategy 2.

d For taxa appearing in Strategy 1 and Strategy 2.

**At the Phylum level,** we found no overlap between the taxa selected by ML-based and network-based methods, highlighting the complementary nature of these approaches. To provide a comprehensive view, we report the most consistently selected taxa within each category.

*ML-based selected taxa: Firmicutes*, identified through ML-based feature selection, comprise 5.5% of the bacterial community and promote plant growth and disease suppression, especially *Bacillus* spp., which enhance root colonization and pathogen inhibition through antimicrobial [74]. *Cyanobacteria* (0.9%) also emerged as important ML-selected taxa, contributing to nitrogen fixation, biofilm formation, and soil structure improvement, benefiting microbial community stability [75]. Less abundant phyla such as *Armatimonadota* (0.2%) may also be directly related to the disease, as negative relationship between the abundance of those phyla and soil suppressive ability of scab has been observed in one of our previous studies [76]. The precise implications of *NB1-j* (0.1%) in disease progression are still unclear, but its involvement in nitrogen cycling and interactions with microalgae suggest potential indirect influences [77].

*Network-based selected taxa (intersection of both strategies): Bacteroidota*, *WPS-2*, and *Proteobacteria* were consistently identified across both network-based strategies, indicating strong and robust association with disease status. *Bacteroidota* (5.5%) are involved in nutrient cycling and pathogen competition, both of which are closely related to disease suppression [78]. Members of the Phylum *Bacteroidota* are plant growth-promoting microbes that are enriched in the rhizosphere, where they enhance plant nutrient uptake and disease suppression [79–81]. The role of *Bacteroidota* in disease suppression is evidenced by their enrichment in suppressive soils [82] and association with improved plant tolerance under pathogen stress [83]. *WPS-2*, though less prevalent (0.3%), showed a negative relationship with suppressive soil capacity in our previous research [76]. *Proteobacteria* represent a taxonomically diverse group containing both beneficial (e.g., *Rhizobium*) and pathogenic (e.g., *Pseudomonas syringae*, *Ralstonia solanacearum*) members, reflecting their complex role in disease ecology [84].

**At the Class level,** *Network-based intersection: Actinobacteria* and *AD3* were selected by both network-based strategies, indicating strong structural importance in microbial networks associated with disease. *Actinobacteria* are key contributors to soil suppressiveness against plant pathogens. Notably, non-pathogenic *Streptomyces* spp. produce antibiotics that inhibit soil-borne pathogens, including *Streptomyces scabies*, the causative agent of common scab disease [85]. Although less well-characterized, *AD3* was identified as a robust Class-level taxon across both network-based strategies. This group has been associated with degraded or polluted soils and reduced organic matter content, suggesting its presence may indicate shifts in microbial community structure linked to soil stress and disease vulnerability [86].

*ML and network-based Strategy 2 intersection: Bacilli*, *Anaerolineae*, and *Ktedonobacteria* were jointly identified by ML-based feature selection and network-based Strategy 2, suggesting these taxa are both predictive and structurally central in the disease-associated microbiome. *Bacilli* (notably *Bacillus* spp.) are widely recognized for their role in plant protection and disease suppression, particularly through *Bacillus* spp., which produce lipopeptides and hydrolytic enzymes that enhance root colonization and pathogen inhibition [87]. The biological significance of *Ktedonobacteria* is unclear; however, studies show that *Ktedonobacteria* exhibit complex morphologies and genomic features, leading to speculation that they may be a valuable microbial resource for novel compounds [88]. *Anaerolineae*, frequently found in low-oxygen soil habitats, play a crucial role in carbon degradation processes, including the breakdown of plant-derived compounds [89, 90]. This activity can modify the soil environment, potentially suppressing plant diseases through nutrient competition or the production of inhibitory substances.

**At the Order level,** *Confirmed by all ML methods and both network-based strategies: C0119* was the only taxon consistently identified by all ML models and both network-based strategies, highlighting its strong and stable association with disease-relevant microbial networks. Although taxonomically unclassified, recent studies have shown that *C0119* is a dominant order in biochar-amended soils, environments known to support improved microbial diversity, carbon cycling, and root-associated community stability [91]. *C0119* has also been shown to respond to various soil health management practices [92], including cover cropping [93] and manure application [94]. The repeated detection of *C0119* across methods and its responsiveness to soil management highlight it as a potential indicator taxon linking microbial community structure with disease outcomes.

*Selected by ML and Network Strategy 1: Defluviicoccales*, while often linked to anaerobic degradation, have been observed in disease-prone soils, where they may contribute to microbial community shifts that influence pathogen persistence [95]. *Bacteroidales* are involved in organic matter degradation and nutrient cycling. Some members have been associated with pathogen suppression via competitive exclusion and enhancement of soil nutrient availability, contributing indirectly to disease resistance [96].

*Selected by ML and Network Strategy 2: Ktedonobacterales* have been associated with disease suppression due to their potential for producing antimicrobial compounds and their metabolic similarity to antibiotic-producing Actinomycetes [88]. Previous studies have also identified *Ktedonobacterales* as keystone taxa in soil microbial networks [97].

## Discussion

This study introduces a comprehensive framework for robust microbiome network inference and the identification of disease-associated microbial taxa, specifically in the context of potato common scab. We developed CMIMN, a novel BN algorithm based on conditional MI, which exhibited superior robustness and interpretability across taxonomic levels. Recognizing the limitations of individual network inference methods, we integrated CMIMN with three widely used approaches, including SPIEC-EASI, SPRING, and SPARCC, to construct consensus microbiome networks. These consensus networks captured biologically meaningful interaction patterns while reducing algorithm-specific variability, thereby enhancing confidence in the inferred microbial interactions. An important limitation of CMIMN is that it does not explicitly correct for compositionality, which is inherent to microbiome abundance data. While the log transformation applied in our implementation improves distributional properties, it does not eliminate compositional constraints and may lead to spurious associations in cases of strong compositional bias. A possible extension of CMIMN is to incorporate compositionality-aware transformations, such as the CLR, as recommended in [98]. Indeed, our implementation allows users to apply CLR prior to network inference by setting quantitative = FALSE and providing CLR-transformed data as input. Another limitation of the current implementation of CMIMN is that it does not preserve the sign of associations (positive vs. negative). By design, we treated all edges as unsigned dependencies, harmonizing methods that can infer signed edges with MI/CMI-based methods (which are inherently unsigned). While this simplifies integration and emphasizes reproducibility, it may obscure ecologically meaningful distinctions between cooperative and competitive interactions. Future work will extend the framework to incorporate signed associations when integrating across multiple inference methods.

We observed differences in F1-scores between the synthetic and bootstrap-based datasets, indicating that data type influences method performance. For instance, some algorithms such as SPRING performed better on synthetic data but showed lower consistency on bootstrap-based datasets. The synthetic datasets were generated using a simulation procedure available in the SPRING framework, and SPRING performed well on this simulated data. Because the bootstrap-based datasets are derived directly from real microbiome data, they better reflect the variability and complexity of biological samples [99]. These results suggest that algorithm performance can vary depending on the characteristics of the dataset and that relying only on synthetic data may give an overly optimistic impression of model robustness.

To identify taxa relevant to disease, we implemented a multi-method feature selection framework combining different ML algorithms with two network-based strategies. In the ML component, although our current approach applies a top-20% cutoff within each method and aggregates presence across multiple algorithms using the TOTAL score, defined as the number of independent ML methods (out of seven) that identified a given OTU as important, this strategy does not explicitly consider the rank position of individual taxa (e.g., ranks 1, 2, or 3). This could result in overlooking taxa that are strongly prioritized by a specific method but do not appear consistently across others. As future work, we plan to avoid aggregating results across ML methods and instead analyze each method independently. By identifying top-ranked taxa from each ML strategy (seven distinct ML methods) and each network-based strategy (multiple centrality and network comparison approaches) separately, and then selecting only those taxa that appear in both groups and are confirmed by multiple methods, while also incorporating their rank positions, we aim to improve the robustness of the final feature set. This approach will allow us to capture both statistical importance and ecological relevance without relying solely on cumulative scoring. Such a dual strategy reduces the risk of overemphasizing taxa that are consistently but only moderately ranked, while highlighting those with stronger overall evidence.

Our results revealed clear distinctions in microbial community structure between “clean tubers” and “scab-infected” tubers. At the Phylum level, *Bacteroidota*, *WPS-2*, and *Proteobacteria* were identified through both network-based strategies, while *Firmicutes* and *Cyanobacteria* were highlighted by ML models. Interactions such as *Actinobacteriota–Proteobacteria* and *Planctomycetota–Patescibacteria* were found to be consistently supported by all four network inference methods and corroborated by existing soil studies, reinforcing their ecological relevance. *Bacteroidota* are widely recognized as key contributors to soil health through organic matter degradation, nutrient cycling, and suppression of soil-borne pathogens. Their abundance has been linked to improved soil quality under practices such as biochar or manure amendment, and they are frequently considered indicators of fertile, resilient soils [100, 101]. The consistent detection of *Bacteroidota* in our potato datasets suggests that this Phylum supports nutrient turnover and contributes to disease suppression in agricultural soils. At the Class level, *Actinobacteria*, *AD3*, *Bacilli*, *Anaerolineae*, and *Ktedonobacteria* were identified by either multiple strategies or the intersection of both ML and network approaches. The Class *Ktedonobacteria* (Phylum *Chloroflexi*) was also detected, but its ecological significance is best reflected by the Order *Ktedonobacterales*, described below. These classes are associated with key ecological functions such as carbon degradation, nutrient cycling, antimicrobial production, and disease suppression. At the Order level, *C0119* was the only taxon confirmed by all ML models and both network-based strategies, highlighting its potential as a robust indicator of disease status. The uncultured order *C0119* (Phylum Chloroflexi) is emerging as an indicator of soil health and management. In paddy soils, its abundance increased significantly following biochar amendment, where it was identified as a dominant group in the rhizosphere, suggesting a strong response to carbon-rich amendments that improve soil fertility and microbial diversity [91]. Similarly, recent work has shown that *C0119* plays an active role in soil carbon metabolism, particularly in the degradation of polysaccharides and cellulose under fertilization and straw-return practices [102]. While its direct role in plant disease suppression remains unclear, the consistent enrichment of C0119 under practices that enhance soil productivity and resilience indicates that it contributes to carbon turnover and may indirectly support disease-suppressive soil environments. Other important orders included *Bacteroidales*, *Defluviicoccales*, and *Ktedonobacterales*, identified by at least two independent methods. *Ktedonobacterales* (Order within the Class *Ktedonobacteria*, Phylum *Chloroflexi*) are increasingly recognized as crucial components of the soil microbiome, particularly for their role in disease suppression through the production of antimicrobial compounds [88]. Their genomes are enriched with diverse biosynthetic gene clusters, enabling the synthesis of secondary metabolites with broad activity against both Gram-positive and Gram-negative bacteria [103]. This biosynthetic potential positions *Ktedonobacterales* as important contributors to the natural defense mechanisms of soil communities, helping to regulate pathogen populations and sustain soil health. Their involvement in disease suppression also highlights them as promising candidates for the discovery of novel bioactive compounds with potential applications in sustainable agriculture [88, 103]. Together, the enrichment of both *C0119* and *Ktedonobacterales*, two distinct lineages within the Phylum *Chloroflexi*, suggests complementary ecological roles, with *C0119* contributing to carbon turnover and nutrient cycling, and *Ktedonobacterales* supporting disease suppression through antimicrobial potential. The topological analysis of microbial networks further revealed differences in connectivity and interaction density between clean and diseased tuber microbiomes. “Clean tuber” networks exhibited higher overall connectivity, suggesting a more stable and cooperative microbial community. In contrast, disease-associated networks were more fragmented and featured shifts in taxa centrality, indicating structural reorganization in response to pathogen pressure. Several interactions identified in these networks, such as *Actinobacteriota–Gemmatimonadota* and *Methylomirabilota–WPS-2*, have also been observed in previous studies investigating soil response to stress or contamination.

## Conclusions

Our findings align with the broader concept of disease-suppressive soils, which highlights fundamental differences between healthy and disease-conducive microbiological environments [104]. Because the microbiomes analyzed here were extracted from preplanting soils, the distinguishing microbial features appear to be present long before disease emergence. Despite variation in geography, management, and climate, the microbial signal remained strong for features associated with disease-free tubers. This underscores the promising utility of soil microbiomes as indicators of soil health and as predictive tools for potato scab risk.

Altogether, our integrative approach provides a scalable and interpretable framework for microbiome network analysis and biomarker discovery. By combining Bayesian inference, consensus-based network construction, and multi-method feature selection, we bridge predictive modeling with ecological insight. These findings not only improve our understanding of microbial community dynamics in disease contexts but also establish a foundation for microbiome-informed strategies in plant health management and sustainable agriculture. As a next step, our broader vision is to develop an interactive Shiny application that enables biologists to upload their microbiome and disease data to identify reliable taxa-disease associations and uncover robust microbial interaction relationships, making advanced analysis tools more accessible and actionable for the biological research community. In addition, our approach extracts biologically meaningful information by comparing network structures between “clean tubers” and “scab-infected tubers”: Strategy 1 focuses on differences in centrality measures across the two networks, while Strategy 2 analyzes each network independently to highlight key taxa. To further enhance this comparative analysis, we are interested in applying the Microbiome Network Alignment (MiNAA) algorithm [105], which aligns microbial networks across conditions, allowing us to extract deeper biological insights about shifts in microbial interactions associated with disease.

An important consideration is the trade-off between stability and discriminative power. Highly stable methods, such as CMIMN, achieved reproducible results with narrow CIs, but this conservatism may also introduce bias, making derived predictions and interpretations less trustworthy. In contrast, less stable approaches (e.g., SE_glasso) produced networks that varied more across bootstrap replicates. While some of these changing relationships may capture biologically meaningful interactions, bootstrap analysis only reflects reproducibility rather than accuracy, where accuracy would mean how well the inferred associations match true underlying biological relationships. Researchers should therefore be aware of these trade-offs, as each approach carries risks that can influence how microbial interactions are inferred.

A limitation of our study is that environmental covariates (e.g., soil chemistry) were not included, so some associations may reflect shared environmental preferences rather than direct interactions. In another study on the same dataset, we demonstrated that environmental features are highly informative for disease prediction [2], but here we focused specifically on microbiome-derived signals. Future work incorporating approaches such as FlashWeave [106], the strategy in [107], or our own framework [108] will help disentangle microbial interactions from environmentally driven patterns.

While our manuscript was under review, a related study was published that also applied a consensus-based approach for microbiome network construction [109]. Our work here represents the first step in a broader framework: we intentionally adopted a simple voting strategy to combine multiple inference methods, providing a transparent and computationally efficient way to capture broad agreement across algorithms. This initial approach allowed us to establish an overall picture of reproducible associations without introducing the added complexity and run-time costs of bootstrap resampling at this stage. In this study, bootstrap analysis was applied separately in the evaluation stage (Section 3.2) to assess the reproducibility of each method’s inferred network, but the consensus itself was based purely on voting. Importantly, our broader framework, CMiNet [25] (R package and Shiny app, under review), extends this approach by integrating ten inference methods with both voting and bootstrap-based reproducibility analysis, providing a more comprehensive solution. Thus, while the present study focuses on demonstrating feasibility and biological application with a streamlined voting approach, our broader research program is advancing toward scalable and reproducible consensus microbiome network construction.

## Supplementary Material

bpaf089_Supplementary_Data

## Data Availability

The 16S and ITS amplicon sequencing data associated with this study are publicly available at the NCBI Short Read Archive under the BioProject PRJNA1135141. The R package of CMIMN and all R code for this paper are available in the github repository, https://github.com/solislemuslab/CMIMN.
