# Supplementary material for: A hybrid framework for disease biomarker discovery in microbiome research combining Bayesian networks, machine learning, and network-based methods
Source: Biol Methods Protoc. 2025 Dec 13;11(1):bpaf089. doi: 10.1093/biomethods/bpaf089 (PMC12791661; doi:10.1093/biomethods/bpaf089)
Supplement: bpaf089_Supplementary_Data [file bpaf089_supplementary_data.pdf]

SUPPLEMENTARY MATERIAL: A HYBRID FRAMEWORK FOR DISEASE BIOMARKER DISCOVERY IN MICROBIOME RESEARCH COMBINING BAYESIAN NETWORKS, MACHINE LEARNING, AND NETWORK-BASED METHODS

List of Figures

S1 Effect of normalization method on `CMIMN` network stability across datasets. . . . 4

S2 Box plots illustrating F1-scores and Jaccard similarities obtained from the robustness analysis of different microbiome network construction methods. . . . 5

S3 Pairwise comparisons of network reproducibility (F1-scores) across inference algorithms. . . . 5

S4 Microbiome network at the Class taxonomic level 6

S5 microbiome network at the Order taxonomic level. . . . 7

S6 Overlap Between Machine Learning Methods based on different nomalized data sets and Network-Based Approaches for 'clean tubers' network 8

S7 Overlap Between Machine Learning Methods based on different nomalized data sets and Network-Based Approaches for 'scab-infected tubers' network 9

S8 Number of taxa selected by ML-based methods across different normalization strategies (CLR, raw count, log, and total-sum scaling) at the Phylum, Class, and Order levels. . . . 14

S9 Number of taxa identified as significant under Strategy 2 across different selection thresholds. . . . 15

List of Tables

S1 Median F1-score and standard deviation (SD) across 50 bootstrap replicates for four normalization methods 3

S2 Median and standard deviation of pairwise F1-scores across 50 bootstrap replicates for networks inferred using different normalization methods. . . . 3

S3 Taxonomic level (first column), number of OTUs in the original dataset (second column), and number of OTUs remaining after filtering out those that appear in fewer than 15 samples (third column). . . . 4

S4 Summary of methods, thresholds, tools, and outputs used in the workflow 10

S5 Mean ± standard deviation (SD) and 95% bootstrap percentile confidence intervals (CI) for F1-scores 10

S6 Pairwise comparison of F1-scores among four network inference methods 11

S7 Performance of four network inference methods on synthetic microbiome data 11

S8 Runtime benchmarks for four network-inference algorithms 12

S9 Network metrics for microbiome networks constructed using four different methods based on all samples at the **Phylum** level. . . . 12

S10 Network metrics for microbiome networks constructed using four different methods based on all samples at the **Class** level. . . . 12

S11 Network metrics for microbiome networks constructed using four different methods based on all samples at the **Order** level. 12

S12 Commonly identified important OTUs based on topological features in microbiome networks constructed from all samples at the **Phylum** level. 13

S13 Commonly identified important OTUs based on topological features in microbiome networks constructed from all samples at the **Class** level. 13

S14 Commonly identified important OTUs based on topological features in microbiome networks constructed from all samples at the **Order** level. 16

S15 Phylum-level microbial associations identified in 'clean tuber' and 'scab-infected tubers' microbiome networks. Only interactions confirmed by all four inference methods are shown. 16

S16 Class-level microbial associations identified in 'clean tuber' and 'scab-infected tubers' microbiome networks. Only interactions confirmed by all four inference methods are shown. 17

S17 Order-level microbial associations identified in 'clean tuber' and 'scab-infected tubers' microbiome networks. Only interactions confirmed by all four inference methods are shown. 17

S18 Important OTUs identified using Multi Machine Learning (ML) methods at the **Phylum** level. 20

S19 Important OTUs identified using Multi Machine Learning (ML) methods at the **Class** level. 21

S20 Important OTUs identified using Multi Machine Learning (ML) methods at the **Order** level. 22

S21 Important OTUs identified as key features in response to pitted scab at the **Phylum** level using Strategy 1: Differential Centrality Analysis. 23

S22 Important OTUs identified as key features in response to pitted scab at the **Class** level using Strategy 1: Differential Centrality Analysis. 23

S23 Important OTUs identified as key features in response to pitted scab at the **Order** level using Strategy 1: Differential Centrality Analysis. 23

S24 Selection of key Operational Taxonomic Units (OTUs) at the **Phylum** level using network-based feature selection (Strategy 2) 24

S25 Selection of key Operational Taxonomic Units (OTUs) at the **Class** level using network-based feature selection (Strategy 2) 25

S26 Selection of key Operational Taxonomic Units (OTUs) at the **Order** level using network-based feature selection (Strategy 2) 27

S27 Selection of Operational Taxonomic Units (OTUs) at the **Phylum** level in both networks of 'Clean Tubers' and 'Scab-Infected Tubers' using Network-Based Method (Strategy 2) and Machine Learning (ML) methods. 31

S28 Selection of Operational Taxonomic Units (OTUs) at the **Class** level in both networks of 'Clean Tubers' and 'Scab-Infected Tubers' using Network-Based Method (Strategy 2) and Machine Learning (ML) methods. 31

S29 Selection of Operational Taxonomic Units (OTUs) at the **Order** level in both networks of 'Clean Tubers' and 'Scab-Infected Tubers' using Network-Based Method (Strategy 2) and Machine Learning (ML) methods. 32

S30 Operational Taxonomic Units (OTUs) of significance in the 'Clean Tubers' network, selected by all four algorithms: `CMIMN, SPARCC, SE_glasso`, and `SPRING`. 33

S31 Operational Taxonomic Units (OTUs) of significance in the 'scab-infected tubers' network, selected by all four algorithms: `CMIMN, SPARCC, SE_glasso`, and `SPRING`. 33

Table S1: Median F1-score and standard deviation across 50 bootstrap replicates for four normalization methods applied before `CMIMN` network inference.

dataset	method	median	standard deviation
Amgut	Log	0.7549505	0.02405883
Amgut	CLR	0.73267327	0.02204017
Amgut	GMPR	0.68440594	0.07197308
Amgut	TSS	0.68069307	0.05366118
Phylum	Log	0.85306815	0.03715509
Phylum	CLR	0.87837838	0.11070246
Phylum	GMPR	0.75675676	0.07881746
Phylum	TSS	0.75675676	0.06363984
Class	Log	0.8369448	0.0274406
Class	CLR	0.84552846	0.03429692
Class	GMPR	0.81707317	0.03778735
Class	TSS	0.8495935	0.02717485
Order	Log	0.86149972	0.01670613
Order	CLR	0.7360179	0.03926627
Order	GMPR	0.79865772	0.0260288
Order	TSS	0.81096197	0.0202064

Table S2: Median and standard deviation of pairwise F1-scores across 50 bootstrap replicates for networks inferred using different normalization methods. Each dataset (Amgut, Phylum, Class, and Order) was analyzed using the four normalization schemes (Log, CLR, GMPR, and TSS), and F1-scores were computed between networks generated under each pair of methods.

dataset	pair	median	standard deviation
Amgut	CLR–GMPR	0.2549505	0.0264617
Amgut	CLR–TSS	0.24133663	0.02186131
Amgut	Log–CLR	0.42326733	0.03907558
Amgut	Log–GMPR	0.41584158	0.02918012
Amgut	Log–TSS	0.42450495	0.02997535
Amgut	GMPR–TSS	0.68688118	0.03210582
Phylum	CLR–GMPR	0.08108108	0.03986007
Phylum	CLR–TSS	0.05405405	0.03572628
Phylum	Log–CLR	0.51351351	0.04634491
Phylum	Log–GMPR	0.13513514	0.06294502
Phylum	Log–TSS	0.13513514	0.05444152
Phylum	GMPR–TSS	0.72972973	0.06687454
Class	CLR–GMPR	0.18495935	0.02150327
Class	CLR–TSS	0.17682927	0.02261023
Class	Log–CLR	0.41463415	0.04328519
Class	Log–GMPR	0.46747968	0.04466459
Class	Log–TSS	0.47154472	0.04344565
Class	GMPR–TSS	0.83943089	0.03812647
Order	CLR–GMPR	0.25391499	0.01741263
Order	CLR–TSS	0.26621924	0.01903195
Order	Log–CLR	0.48657718	0.01914406
Order	Log–GMPR	0.45973154	0.0280264
Order	Log–TSS	0.47203579	0.02915139
Order	GMPR–TSS	0.84619686	0.03204466

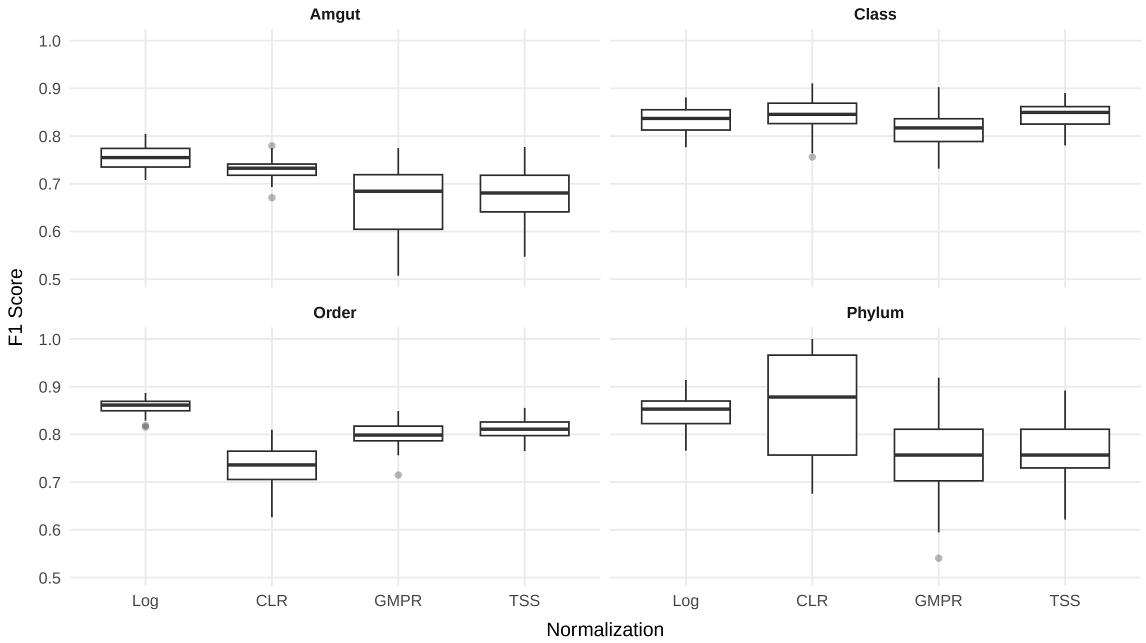

Figure S1: Effect of normalization method on CMIMN network stability across datasets. Boxplots show the distribution of F1-scores obtained from 50 bootstrap replicates under four normalization strategies: logarithmic (Log), Centered Log-Ratio (CLR), Geometric Mean of Pairwise Ratios (GMPR), and Total Sum Scaling (TSS). Analyses were performed on four datasets representing different taxonomic levels—Phylum, Class, Order—and the amgut1 dataset from the American Gut Project (Amgut). Higher F1-scores indicate greater reproducibility of inferred networks.

Table S3: Taxonomic level (first column), number of OTUs in the original dataset (second column), and number of OTUs remaining after filtering out those that appear in fewer than 15 samples (third column).

Level	# OTUs	# OTUs after filtering
Phylum	57	38
Class	152	99
Order	378	189

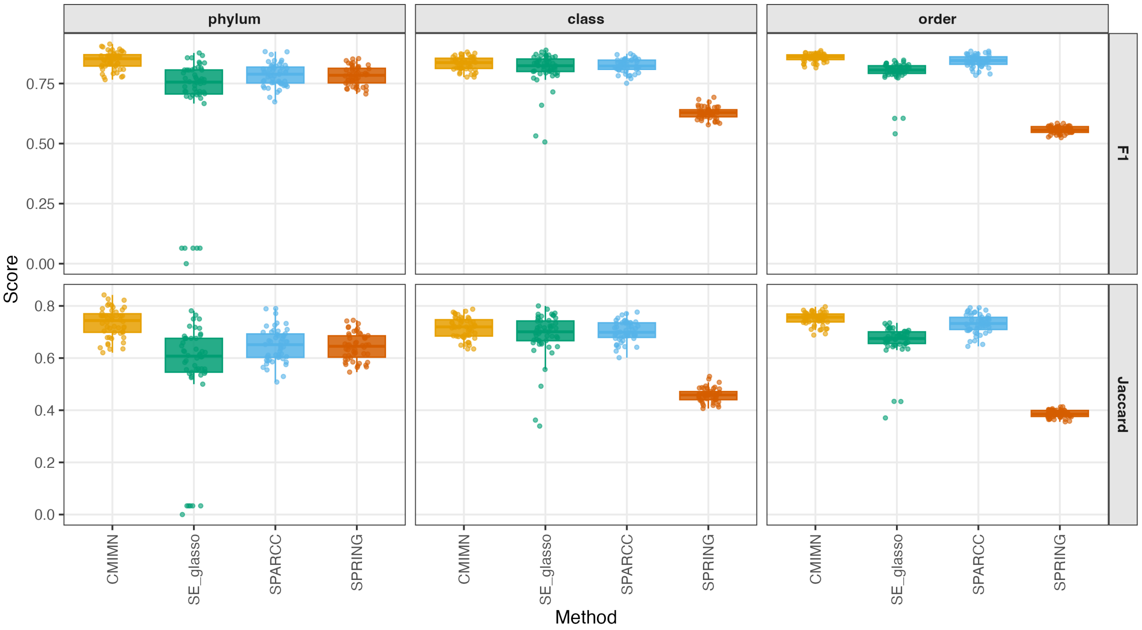

Figure S2: Robustness of microbial network inference algorithms across taxonomic levels. Box plots summarize the distribution of (top) F1-scores and (bottom) Jaccard similarities from 50 bootstrap replicates for four methods (`CMIMN`, `SE_glasso`, `SPARCC`, and `SPRING`) at the Phylum, Class, and Order levels. The F1-score quantifies agreement between bootstrap-derived networks and the full-sample reference network by balancing precision and recall, while the Jaccard index measures the proportion of shared edges between networks. Higher values in both metrics indicate more reproducible network structures. Across taxonomic levels, `CMIMN` and `SPARCC` exhibited consistently high F1 and Jaccard values, whereas `SE_glasso` and particularly `SPRING` displayed lower scores and greater variability, especially at finer taxonomic resolution.

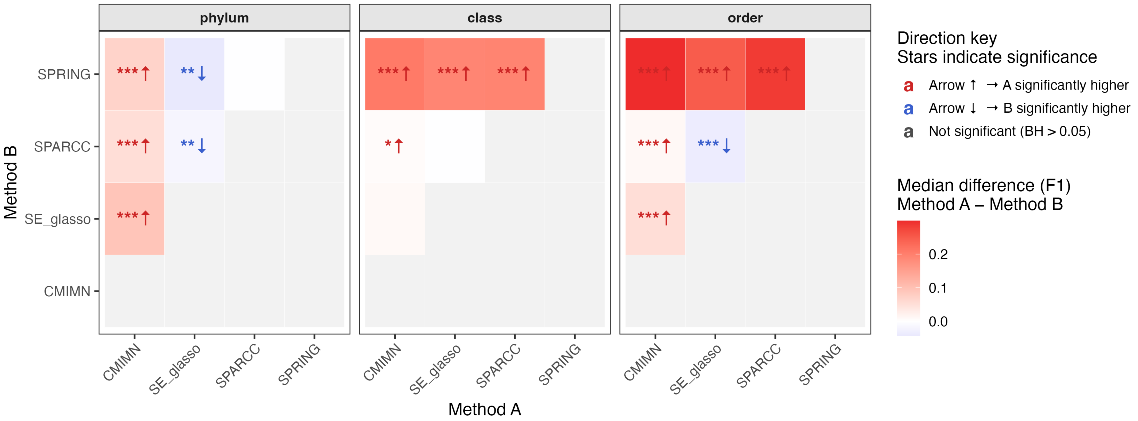

Figure S3: Pairwise comparisons of network reproducibility (F1-scores) across inference algorithms. Heatmaps show the median difference in F1-score (Method A – Method B) from 50 bootstrap replicates at the Phylum, Class, and Order levels. Red tiles indicate that Method A achieved higher median F1-scores than Method B, whereas blue tiles indicate the opposite. Stars denote statistical significance based on the Wilcoxon signed-rank test with Benjamini–Hochberg correction (* $p \leq 0.05$, ** $p \leq 0.01$, *** $p \leq 0.001$), and arrows indicate the direction of the difference. Blank diagonal tiles represent self-comparisons, which are not evaluated. Across all taxonomic levels, `CMIMN` consistently outperformed `SPRING` with strong significance, while differences between `CMIMN` and `SPARCC` were smaller and less consistent.

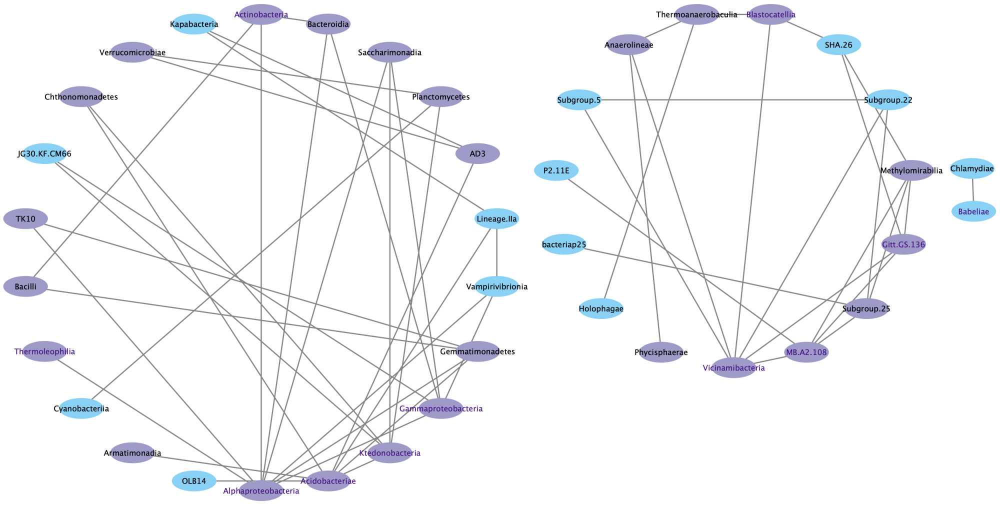

'clean tubers' network

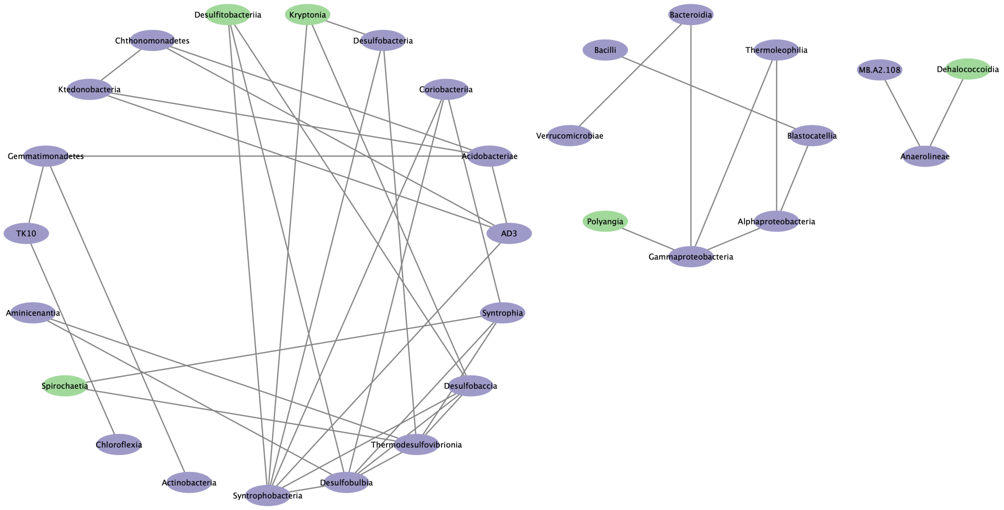

'scab-infected tubers' network

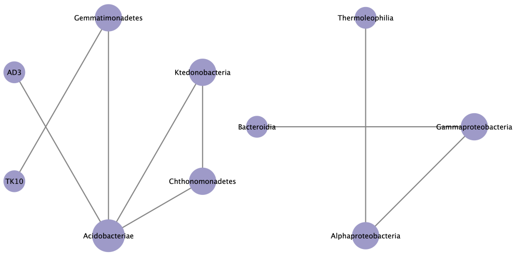

common interactions

Figure S4: This figure illustrates the consensus microbiome network at the Class taxonomic level. Part (a) represents the 'clean tubers' network, part (b) displays the 'scab-infected tubers' network, and part (c) shows the common interactions between them. Nodes correspond to Operational Taxonomic Units (OTUs) and are color-coded: purple for OTUs shared between 'clean tubers' and 'scab-infected tubers' networks, blue for OTUs unique to the 'clean tubers' network, and green for OTUs unique to the 'scab-infected tubers' network. Edges are shown by solid lines which confirmed by all four method).

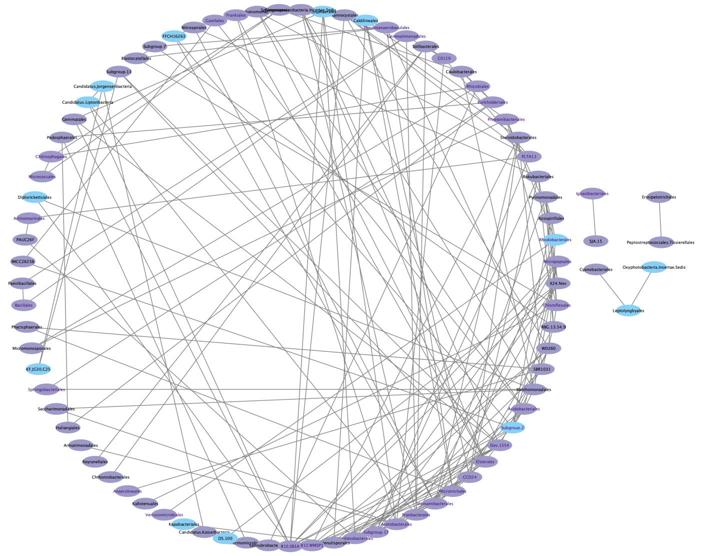

'clean tubers' network

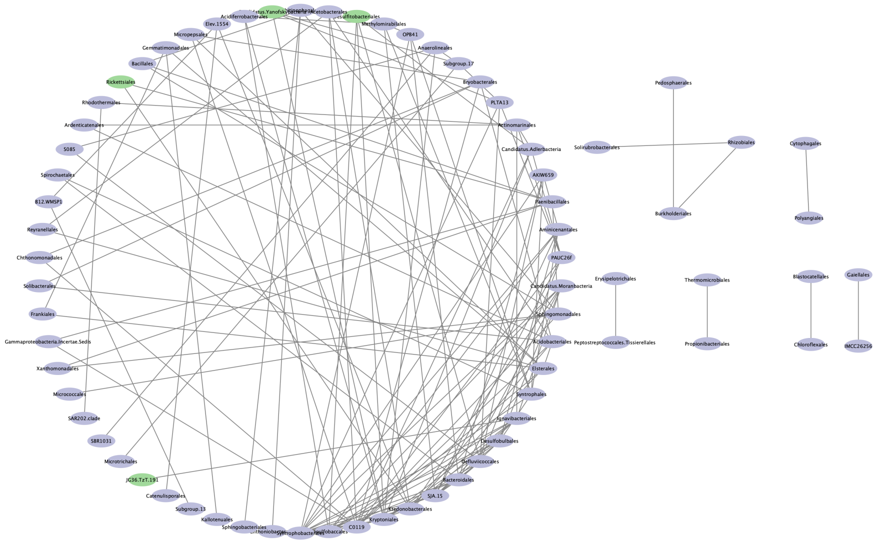

'scab-infected tubers' network

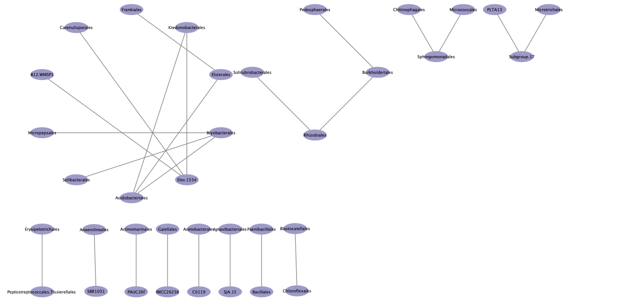

common interactions

Figure S5: This figure showcases the microbiome network at the Order taxonomic level. Part (a) represents the 'clean tubers' network, part (b) displays the 'scab-infected tubers' network, and part (c) shows the common interactions between them. Nodes correspond to Operational Taxonomic Units (OTUs) and are color-coded: purple for OTUs shared between 'clean tubers' and 'scab-infected tubers' networks, blue for OTUs unique to the 'clean tubers' network, and green for OTUs unique to the 'scab-infected tubers' network. The top 20% of nodes with the highest centrality scores resulted by Equation (5) are labeled in dark purple. Node size reflects their degree of connectivity. Edges are shown as solid lines that confirmed by all four methods.

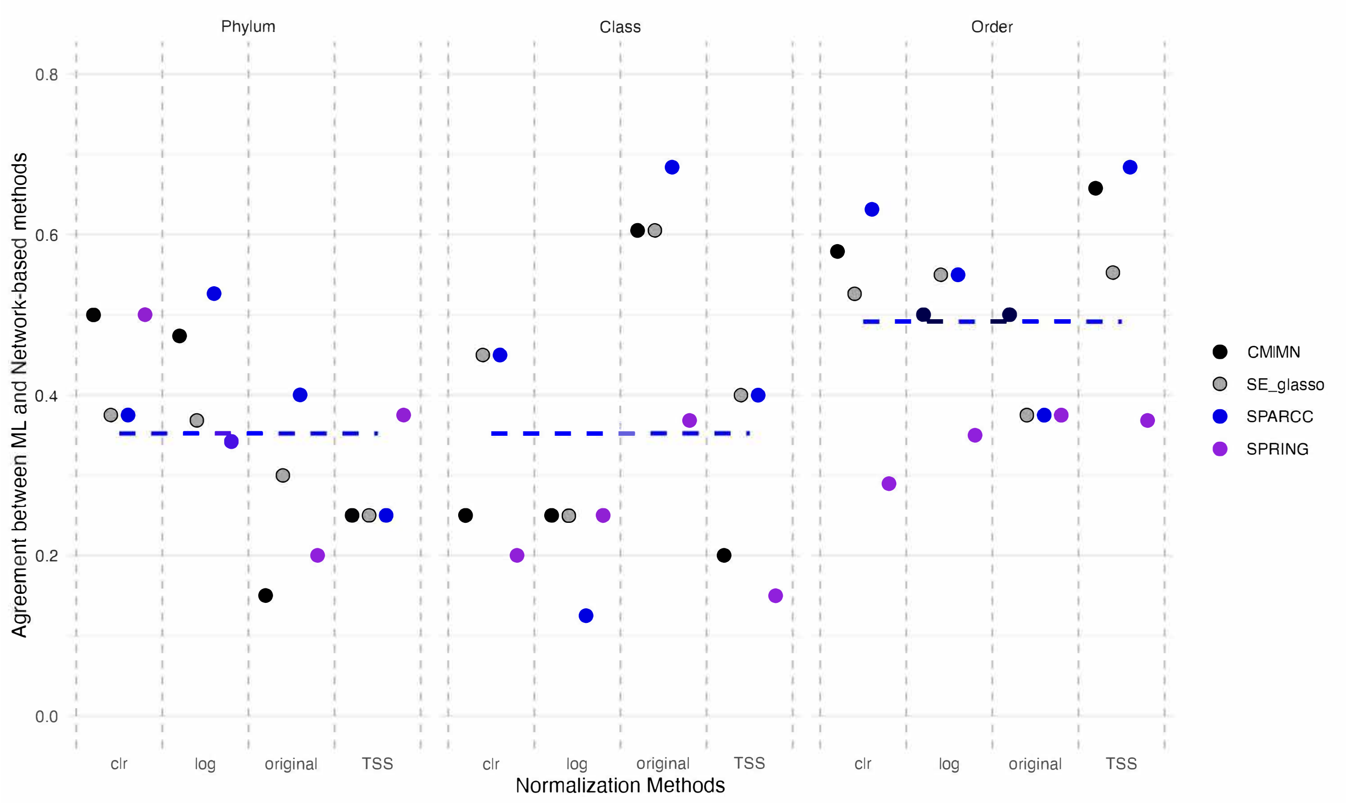

Figure S6: Average overlap between Machine Learning (ML) methods and network-based approaches for the 'clean tubers' network at Phylum, Class, and Order levels, based on four normalization strategies (CLR, log transformation, original, and TSS). Each point represents the agreement between ML-based and network-based methods for a specific normalization and taxonomic level. The dashed horizontal line indicates the overall mean agreement across all methods and normalization strategies, serving as a reference point for comparison.

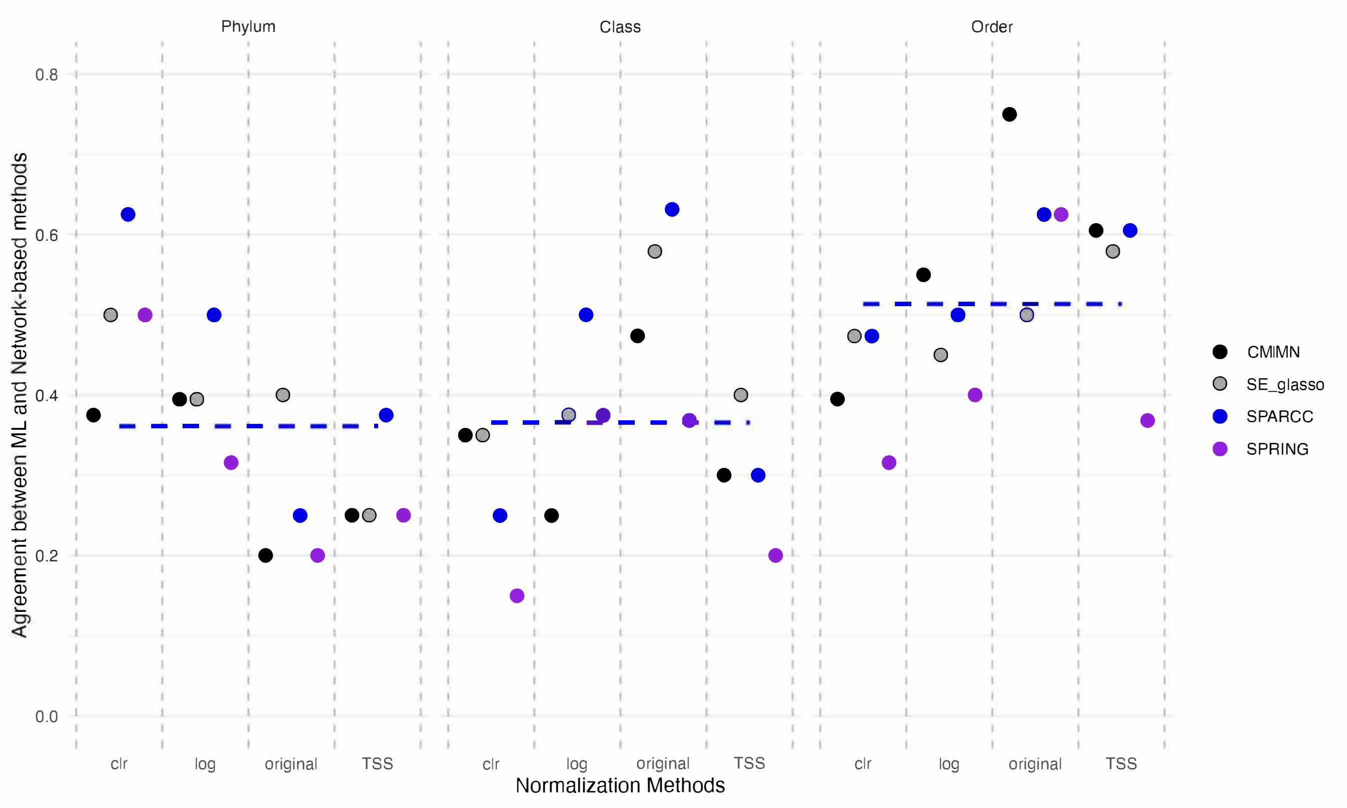

Figure S7: Average overlap between Machine Learning (ML) methods and network-based approaches for the 'scab-infected tubers' network at Phylum, Class, and Order levels, based on four normalization strategies (CLR, log transformation, original, and TSS). Each point represents the agreement between ML-based and network-based methods for a specific normalization and taxonomic level. The dashed horizontal line indicates the overall mean agreement across all methods and normalization strategies, serving as a reference point for comparison.

Table S4: Summary of methods, thresholds, tools, and outputs used in the workflow (corresponding to Figure 1).

**Step**	**Methods**	**Thresholds / Criteria**	**Coding language/Packages**	**Output**
Data Preparation	Filter abundance tables at Phylum, Class, and Order	Remove OTUs present in fewer than 15 samples	R	Filtered abundance tables at multiple taxonomic levels
Network Construction	CMIMN (ours)<br>SPARCC<br>SE_glasso<br>SPRING	Apply log transformation before running CMIMN	CMIMN, SpiecEasi, and SPRING (R packages); SPARCC (R scripts)	Microbiome networks at each taxonomic level, inferred using each method
Feature Selection (ML)	SelectKBest (KBest)<br>Logistic Regression (LR)<br>Decision Tree (DT)<br>Gradient Boosting (GB)<br>Random Forest (RF)<br>Mutual Information<br>Select top 20% of OTUs with the highest scores)	Normalize data (CLR, log, filtered, TSS), before using ML methods<br><br>Select Top 20% of OTUs (highest scores)	Python (scikit-learn)	Ranked list of important OTUs based on TOTAL score (number of ML methods confirming each taxon as important)
Feature Selection (Network-based)	Centrality metrics: Degree, Betweenness, Closeness, Eigenvector, PageRank	Top 20% of OTUs with largest centrality differences between conditions	R	Ranked list of OTUs selected by network-based methods

Table S5: Mean ± standard deviation (SD) and 95% bootstrap percentile confidence intervals (CI) for F1-scores across taxonomic levels and network inference algorithms. level: taxonomic rank at which network performance was evaluated (phylum, class, order). algorithm: the network inference method used (CMIMN, SE_glasso, SPRING, SPARCC). mean_sd: average F1-score ± SD across bootstrap replicates, indicating the central tendency and variability of performance. ci_interval: 95% bootstrap percentile CI $(\theta^*_{0.025}, \theta^*_{0.975})$, representing the range of F1-scores expected due to sampling variability.

level	algorithm	mean_sd	ci_interval
Phylum	CMIMN	0.846 ± 0.037	[0.776, 0.904]
Phylum	SE_glasso	0.682 ± 0.240	[0.065, 0.865]
Phylum	SPRING	0.782 ± 0.038	[0.722, 0.851]
Phylum	SPARCC	0.785 ± 0.047	[0.697, 0.879]
Class	CMIMN	0.833 ± 0.027	[0.779, 0.876]
Class	SE_glasso	0.810 ± 0.073	[0.561, 0.879]
Class	SPRING	0.628 ± 0.024	[0.585, 0.681]
Class	SPARCC	0.824±0.027	[0.773, 0.870]
Order	CMIMN	0.857 ± 0.017	[0.821, 0.880]
Order	SE_glasso	0.796 ± 0.058	[0.605, 0.844]
Order	SPRING	0.557 ± 0.015	[0.529, 0.583]
Order	SPARCC	0.845 ± 0.024	[0.791, 0.883]

Table S6: Pairwise comparison of F1-scores among four network inference methods (CMIMN, SPARCC, SE_glasso, and SPRING) across three taxonomic levels (Phylum, Order, Class). For each pair of methods, the median F1-scores, their differences, and Wilcoxon signed-rank test p-values are reported. Adjusted p-values were computed using the Benjamini–Hochberg correction (p_adj_BH). Significant differences (p_adj_BH < 0.05) indicate that the median F1-scores between two methods differ significantly. In nearly all comparisons, CMIMN achieved higher median F1-scores than other methods, with especially strong improvements observed at the order and class levels.

level	method_A	method_B	A_median	B_median	median_diff	wilcox_p	p_adj_BH
Phylum	CMIMN	SE_glasso	0.85306815	0.75583706	0.09278666	3.68E-09	1.66E-08
Phylum	CMIMN	SPRING	0.85306815	0.78431988	0.06974769	5.52E-09	1.66E-08
Phylum	CMIMN	SPARCC	0.85306815	0.78889743	0.053103	1.10E-08	2.20E-08
Phylum	SE_glasso	SPARCC	0.75583706	0.78889743	-0.0186849	0.00333963	0.00500945
Phylum	SE_glasso	SPRING	0.75583706	0.78431988	-0.0427266	0.00707561	0.00849073
Phylum	SPARCC	SPRING	0.78889743	0.78431988	-0.0015635	0.83181673	0.83181673
Class	CMIMN	SPRING	0.8369448	0.62890173	0.20862194	7.79E-10	2.34E-09
Class	SPARCC	SPRING	0.82273902	0.62890173	0.19415497	7.79E-10	2.34E-09
Class	SE_glasso	SPRING	0.82407407	0.62890173	0.19170619	1.19E-09	2.38E-09
Class	CMIMN	SPARCC	0.8369448	0.82273902	0.00698082	0.02449889	0.03674833
Class	CMIMN	SE_glasso	0.8369448	0.82407407	0.00976703	0.05978317	0.07173981
Class	SE_glasso	SPARCC	0.82407407	0.82273902	-0.0016596	0.85447453	0.85447453
Order	CMIMN	SE_glasso	0.86149972	0.80593125	0.05252039	7.79E-10	9.93E-10
Order	CMIMN	SPRING	0.86149972	0.55651604	0.29925067	7.79E-10	9.93E-10
Order	SE_glasso	SPARCC	0.80593125	0.84563331	-0.0356982	7.79E-10	9.93E-10
Order	SPARCC	SPRING	0.84563331	0.55651604	0.28636146	7.79E-10	9.93E-10
Order	SE_glasso	SPRING	0.80593125	0.55651604	0.24960472	8.28E-10	9.93E-10
Order	CMIMN	SPARCC	0.86149972	0.84563331	0.01104641	2.80E-05	2.80E-05

Table S7: Performance of four network inference methods on synthetic microbiome data generated from a band-structured ground truth network. For each method, the inferred adjacency matrix was compared against the true network using precision, recall, and F1-score. Parameters of each algorithm were tuned so that the number of inferred edges was comparable to the true network.

Method	TP	FP	FN	Edges (est.)	Edges (true)	Precision	Recall	F1
SE_glasso	151	52	24	406	350	0.74	0.86	0.79
SPRING	170	17	5	374	350	0.91	0.97	0.94
SparCC	122	53	53	350	350	0.70	0.70	0.70
CMIMN	161	81	14	484	350	0.67	0.92	0.77

Table S8: Runtime benchmarks for four network-inference algorithms on the same dataset of n = 214 samples with taxon counts varying by level (38/99/189 for phylum/class/order). memory was not profiled. Benchmarks were run on workstation using 8 CPU cores and 16 GB of RAM..

level	n_taxa	algorithm	time_sec
phylum	38	SPARCC	0.205
phylum	38	SE_glasso	8.276
phylum	38	SPRING	28.681
phylum	38	CMIMN	0.139
class	99	SPARCC	0.936
class	99	SE_glasso	23.247
class	99	SPRING	213.666
class	99	CMIMN	1.336
order	189	SPARCC	4.211
order	189	SE_glasso	177.559
order	189	SPRING	862.286
order	189	CMIMN	9.052

Table S9: Network metrics for microbiome networks constructed using four different methods based on all samples at the **Phylum** level.

Method	num_edges	average_path_length	transitivity	mean_degree	mean_distance	modularity
SPARCC	148	1.917	0.506	3.895	1.917	0.238
SE_glasso	60	2.322	0.429	1.579	2.322	0.351
SPRING	172	2.658	0.272	4.526	2.658	0.456
CMIMN	136	3.06	0.636	3.579	3.06	0.372

Table S10: Network metrics for microbiome networks constructed using four different methods based on all samples at the **Class** level.

topology_name	num_edges	average_path_length	transitivity	mean_degree	mean_distance	modularity
SPARCC	880	2.249	0.597	8.889	2.249	0.195
SE_glasso	228	2.388	0.562	2.303	2.388	0.333
SPRING	732	2.697	0.208	7.394	2.697	0.409
CMIMN	856	2.402	0.603	8.646	2.402	0.467

Table S11: Network metrics for microbiome networks constructed using four different methods based on all samples at the **Order** level.

topology_name	num_edges	average_path_length	transitivity	mean_degree	mean_distance	modularity
SPARCC	3400	2.091	0.647	17.989	2.091	0.212
SE_glasso	2170	2.414	0.503	11.481	2.414	0.324
SPRING	2060	2.595	0.175	10.899	2.595	0.399
CMIMN	3574	2.391	0.626	18.91	2.391	0.426

Table S12: Commonly identified important OTUs based on topological features in microbiome networks constructed from all samples at the **Phylum** level. Microbiome networks were constructed using four different inference methods: `SE_glasso`, `SPRING`, `SPARCC`, and `CMIMN`. For each network, key topological features—Degree, Betweenness, Closeness, Eigenvector Centrality, and PageRank—were computed to assess the importance of individual OTUs. The top 20% of OTUs based on each centrality measure were selected as important. An OTU was included in this table if it consistently ranked in the top 20% across all four network inference methods based on at least one topological feature. For example, *Proteobacteria* was ranked in the top 20% of networks generated by all four methods based on Degree, Closeness, and PageRank.

Important OTUs	Features
Proteobacteria	Degree, Closeness, Page Rank
Acidobacteriota	Degree, Closeness, Page Rank
WPS.2	Betweenness, Closeness
NB1.j	Page Rank

Table S13: Commonly identified important OTUs based on topological features in microbiome networks constructed from all samples at the **Class** level. Microbiome networks were constructed using four different inference methods: `SE_glasso`, `SPRING`, `SPARCC`, and `CMIMN`. For each network, key topological features—Degree, Betweenness, Closeness, Eigenvector Centrality, and PageRank—were computed to assess the importance of individual OTUs. The top 20% of OTUs based on each centrality measure were selected as important. An OTU was included in this table if it consistently ranked in the top 20% across all four network inference methods based on at least one topological feature. For example, *Acidobacteriae* was consistently ranked in the top 20% of networks generated by all four methods based on all topological features.

**OTUs**	**Topology Measures**
Acidobacteriae	Degree, Betweenness, Closeness, Eigenvector Centrality, Page Rank
Alphaproteobacteria	Degree, Betweenness, Closeness, Page Rank
Anaerolineae	Degree, Betweenness, Closeness
Ignavibacteria	Degree, Eigenvector Centrality, Page Rank
Ktedonobacteria	Betweenness
Gammaproteobacteria	Betweenness, Page Rank
MB.A2.108	Closeness
OM190	Closeness
Syntrophobacteria	Eigenvector Centrality
Kryptonia	Eigenvector Centrality
Desulfobulbia	Eigenvector Centrality

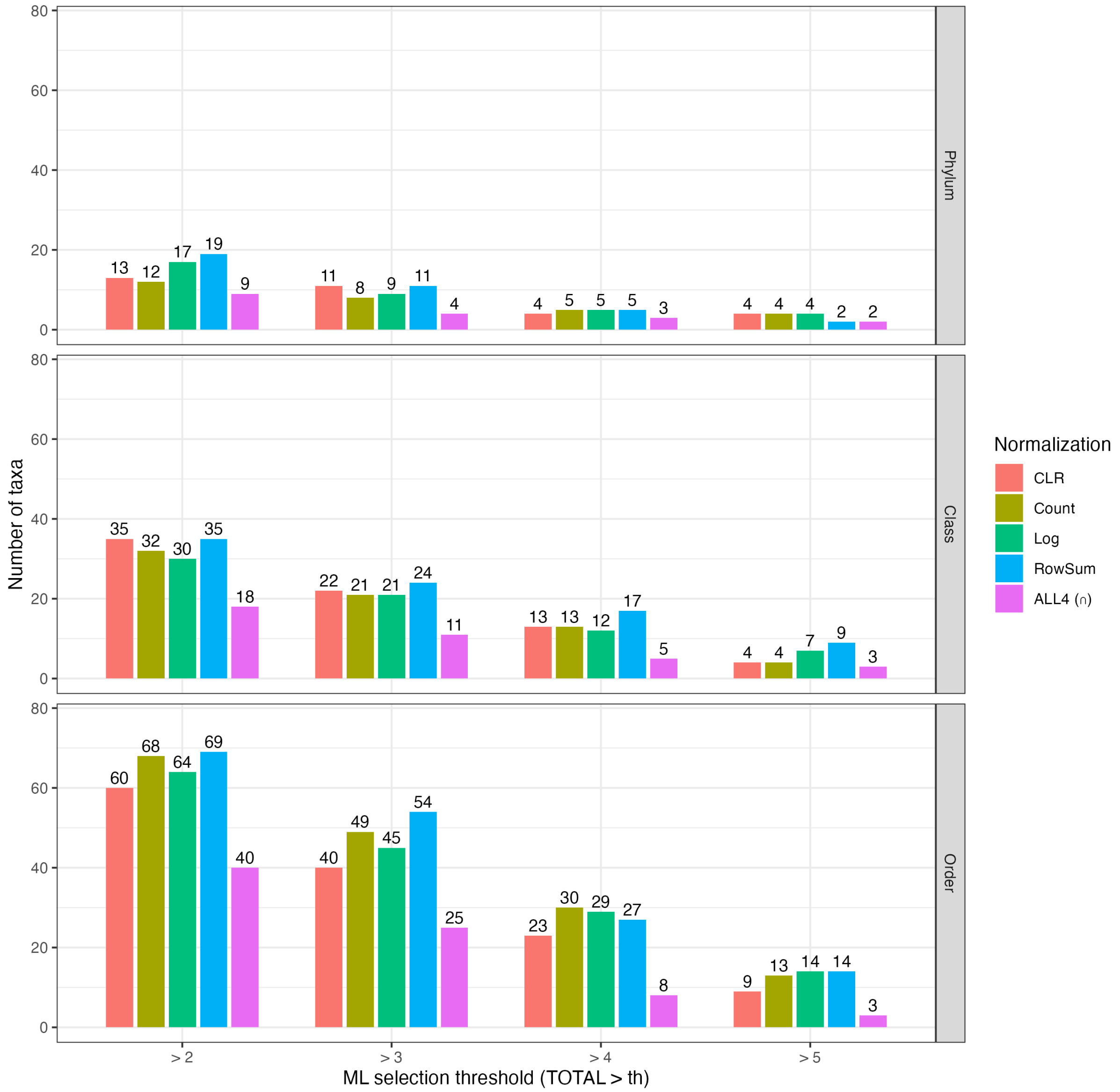

Figure S8: Number of taxa selected by ML-based methods across different normalization strategies (CLR, raw counts, log, and total-sum scaling) at the Phylum, Class, and Order levels. Bars indicate the total number of OTUs selected above different consensus thresholds (TOTAL > 2, > 3, > 4, > 5), where the TOTAL score represents the number of ML algorithms in which each OTU was identified as important. Consistent patterns across normalization methods indicate stable and reproducible ML feature selection. The threshold TOTAL > 3 (i.e., supported by four or more ML approaches) was adopted as the selection cutoff, where 4, 11, and 25 taxa were consistently identified across all normalization methods at the Phylum, Class, and Order levels, respectively. The names of these taxa are provided in Table 1 of the main paper. This threshold is not a hyper-parameter of any algorithm but rather a practical criterion used to define the subset of top taxa for visualization and comparison across selection strategies.

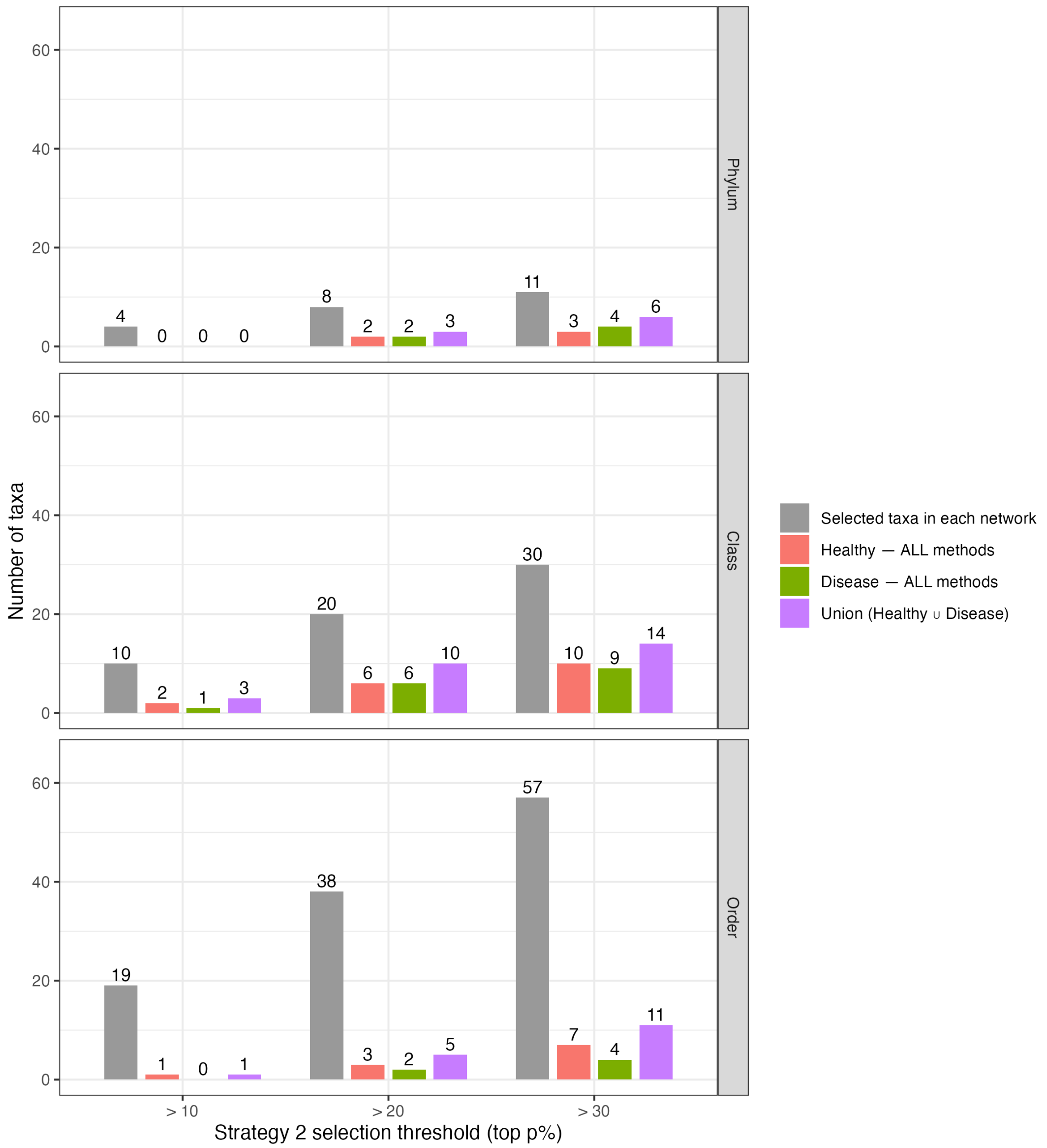

Figure S9: Number of taxa identified as significant under Strategy 2 (composite scoring approach) across different selection thresholds (top 10 %, 20 %, and 30 %). Each row corresponds to one taxonomic level (Phylum, Class, and Order). The first bar shows the total number of taxa identified within each network (healthy or diseased) based on each network-inference algorithm. This number is identical across methods because the top p % of taxa were selected in each case. The second bar indicates the number of OTUs consistently selected in the healthy (clean tubers) network by all four algorithms (i.e., the intersection of important taxa identified by CMIMN, SPARCC, SPRING, and SE_glasso). The third bar shows the number of OTUs confirmed in the disease (scab-infected tubers) network by all four methods. The fourth bar displays the number of OTUs shared between the healthy and diseased networks, representing the final robust set of taxa identified under Strategy 2. We adopted the top 20 % threshold as the cutoff for selecting significant taxa, and the names of these taxa are provided in Table 1 of the main text.

Table S14: Commonly identified important OTUs based on topological features in microbiome networks constructed from all samples at the **Order** level. Microbiome networks were constructed using four different inference methods: `SE_glasso`, `SPRING`, `SPARCC`, and `CMIMN`. For each network, key topological features—Degree, Betweenness, Closeness, Eigenvector Centrality, and PageRank—were computed to assess the importance of individual OTUs. The top 20% of OTUs based on each centrality measure were selected as important. An OTU was included in this table if it consistently ranked in the top 20% across all four network inference methods based on at least one topological feature. For example, *C0119* was consistently ranked in the top 20% of networks generated by all four methods based on all topological features.

**OTUs**	**Topology Measures**
C0119	Degree, Betweenness, Closeness, Eigenvector Centrality, Page Rank
Sphingomonadales	Degree, Closeness, Page Rank
Gammaproteobacteria.Incertae	Degree, Betweenness, Closeness, Page Rank
Defluviicoccales	Degree, Page Rank
Microtrichales	Degree, Page Rank
Gemmatimonadales	Betweenness
Propionibacteriales	Betweenness
B10.SB3A	Betweenness
Erysipelotrichales	Closeness
Reyranellales	Closeness

Table S15: Phylum-level microbial associations identified in 'clean tuber' and 'scab-infected tubers' microbiome networks. Only interactions confirmed by all four inference methods are shown.

**'clean tubers' Network Links**	**'scab-infected tubers' Network Links**	**Common Links**
Chloroflexi–Acidobacteriota	Actinobacteriota–Gemmatimonadota	Actinobacteriota–Proteobacteria
Chloroflexi–Planctomycetota	Actinobacteriota–Acidobacteriota	Proteobacteria–Acidobacteriota
Gemmatimonadota–Acidobacteriota	Gemmatimonadota–Acidobacteriota	
Gemmatimonadota–Proteobacteria	Proteobacteria–Firmicutes	
Planctomycetota–Patescibacteria	Proteobacteria–Myxococcota	
Methylomirabilota–WPS.2	Bacteroidota–Patescibacteria	
Armatimonadota–WPS.2	NB1.j–MBNT15	
Proteobacteria–Bacteroidota	Spirochaetota–MBNT15	
	Desulfobacterota–MBNT15	
	Verrucomicrobiota–Bacteroidota	
	Myxococcota–Bacteroidota	

Table S16: Class-level microbial associations identified in 'clean tuber' and 'scab-infected tubers' microbiome networks. Only interactions confirmed by all four inference methods are shown.

Clean Network Links	Disease Network Links	Common Links
AD3–Kapabacteria	AD3–Chthonomonadetes	AD3–Acidobacteriae
AD3–Verrucomicrobiae	AD3–Ktedonobacteria	Acidobacteriae–Chthonomonadetes
Acidobacteriae–Armatimonadia	AD3–Syntrophobacteria	Acidobacteriae–Gemmatimonadetes
Acidobacteriae–Lineage.IIa	Actinobacteria–Gemmatimonadetes	Acidobacteriae–Ktedonobacteria
Acidobacteriae–OLB14	Alphaproteobacteria–Blastocatellia	Alphaproteobacteria–Gammaproteobacteria
Actinobacteria–Alphaproteobacteria	Aminicenantia–Desulfobulbia	Alphaproteobacteria–Thermoleophilia
Actinobacteria–Bacilli	Aminicenantia–Thermodesulfovibrionia	Bacteroidia–Gammaproteobacteria
Actinobacteria–Bacteroidia	Anaerolineae–Dehalococcoidia	Chthonomonadetes–Ktedonobacteria
Alphaproteobacteria–Bacteroidia	Anaerolineae–MB.A2.108	Gemmatimonadetes–TK10
Alphaproteobacteria–Gemmatimonadetes	Bacilli–Blastocatellia	
Alphaproteobacteria–Saccharimonadia	Bacteroidia–Verrucomicrobiae	
Alphaproteobacteria–TK10	Chloroflexia–TK10	
Alphaproteobacteria–Vampirivibrionia	Coriobacteriia–Desulfobulbia	
Anaerolineae–Phycisphaerae	Coriobacteriia–Syntrophia	
Anaerolineae–Thermoanaerobaculia	Coriobacteriia–Syntrophobacteria	
Anaerolineae–Vicinamibacteria	Desulfitobacteriia–Desulfobaccia	
Babeliae–Chlamydiae	Desulfitobacteriia–Desulfobulbia	
Bacilli–Gemmatimonadetes	Desulfitobacteriia–Syntrophobacteria	
Blastocatellia–SHA.26	Desulfobaccia–Desulfobulbia	
Blastocatellia–Thermoanaerobaculia	Desulfobaccia–Kryptonia	
Blastocatellia–Vicinamibacteria	Desulfobaccia–Syntrophobacteria	
Cyanobacteriia–Planctomycetes	Desulfobaccia–Thermodesulfovibrionia	
Gammaproteobacteria–JG30.KF.CM66	Desulfobacteria–Kryptonia	
Gammaproteobacteria–Saccharimonadia	Desulfobacteria–Syntrophobacteria	
Gammaproteobacteria–Vampirivibrionia	Desulfobacteria–Thermodesulfovibrionia	
Gitt.GS.136–MB.A2.108	Desulfobulbia–Syntrophia	
Gitt.GS.136–Methylomirabilia	Desulfobulbia–Syntrophobacteria	
Gitt.GS.136–SHA.26	Desulfobulbia–Thermodesulfovibrionia	
Gitt.GS.136–Vicinamibacteria	Gammaproteobacteria–Polyangia	
Holophagae–Thermoanaerobaculia	Gammaproteobacteria–Thermoleophilia	
JG30.KF.CM66–Ktedonobacteria	Kryptonia–Syntrophobacteria	
Kapabacteria–Lineage.IIa	Spirochaetia–Syntrophia	
Ktedonobacteria–Planctomycetes	Spirochaetia–Thermodesulfovibrionia	
Ktedonobacteria–Saccharimonadia	Syntrophia–Thermodesulfovibrionia	
Lineage.IIa–Vampirivibrionia		
MB.A2.108–Methylomirabilia		
MB.A2.108–P2.11E		
MB.A2.108–Subgroup.25		
MB.A2.108–Vicinamibacteria		
Methylomirabilia–SHA.26		
Methylomirabilia–Subgroup.25		
Planctomycetes–Verrucomicrobiae		
Subgroup.22–Subgroup.25		
Subgroup.22–Subgroup.5		
Subgroup.22–Vicinamibacteria		
Subgroup.25–bacteriap25		
Subgroup.5–Vicinamibacteria		

Table S17: Order-level microbial associations identified in 'clean tuber' and 'scab-infected tubers' microbiome networks. Only interactions confirmed by all four inference methods are shown.

Clean Network Links	Disease Network Links	Common Links
Acetobacterales–Armatimonadales	AKIW659–Aminicenantales	Acetobacterales–C0119
Acetobacterales–B12.WMSP1	AKIW659–Bacteroidales	Acidobacteriales–Bryobacterales
Acetobacterales–Bryobacterales	AKIW659–Defluviicoccales	Acidobacteriales–Elsterales
Acetobacterales–Elsterales	AKIW659–Syntrophales	Acidobacteriales–Ktedonobacterales
Acetobacterales–Frankiales	AKIW659–Syntrophobacterales	Actinomarinales–PAUC26f
Acetobacterales–Isosphaerales	Acetobacterales–Acidobacteriales	Anaerolineales–SBR1031
Acetobacterales–Saccharimonadales	Acetobacterales–Ktedonobacterales	B12.WMSP1–Elev.1554
Acidobacteriales–Chthonomonadales	Acetobacterales–Reyranellales	Bacillales–Paenibacillales
Acidobacteriales–Gaiellales	Acidiferrobacterales–Desulfobaccales	Blastocatellales–Chloroflexales

Continued on next page

Table S17 Continued from previous page

Clean Network Links	Disease Network Links	Common Links
Acidobacteriales–Micropepsales	Acidiferrobacterales–Kryptoniales	Bryobacterales–Micropepsales
Acidobacteriales–Subgroup.2	Acidiferrobacterales–Methylomirabilales	Bryobacterales–Solibacterales
Acidobacteriales–Xanthomonadales	Acidobacteriales–Ardenticatenales	Burkholderiales–Pedosphaerales
Actinomarinales–PLTA13	Acidobacteriales–C0119	Burkholderiales–Rhizobiales
Azospirillales–Chloroflexales	Acidobacteriales–Solibacterales	Catenulisporales–Elev.1554
Azospirillales–Propionibacteriales	Actinomarinales–Ardenticatenales	Chitinophagales–Sphingomonadales
Azospirillales–Pyrinomonadales	Actinomarinales–Candidatus.Adlerbacteria	Elev.1554–Ktedonobacterales
Azospirillales–Rhodobacterales	Actinomarinales–Ignavibacteriales	Elsterales–Frankiales
Azospirillales–Subgroup.2	Actinomarinales–Rhodothermales	Erysipelotrichales–Peptostreptococcales.Tissierellales
B10.SB3A–B12.WMSP1	Aminicenantales–Desulfobaccales	Gaiellales–IMCC26256
B10.SB3A–Candidatus.Liptonbacteria	Aminicenantales–Desulfobulbales	Ignavibacteriales–SJA.15
B10.SB3A–Catenulisporales	Aminicenantales–SJA.15	Micrococcales–Sphingomonadales
B10.SB3A–Diplorickettsiales	Aminicenantales–Syntrophales	Microtrichales–Subgroup.17
B10.SB3A–Elev.1554	Aminicenantales–Syntrophobacterales	PLTA13–Subgroup.17
B10.SB3A–Gemmatales	Anaerolineales–Kryptoniales	Rhizobiales–Solirubrobacterales
B10.SB3A–Isosphaerales	Anaerolineales–S085	
B10.SB3A–Ktedonobacterales	Anaerolineales–Subgroup.17	
B10.SB3A–Micropepsales	B12.WMSP1–Subgroup.13	
B10.SB3A–Nannocystales	Bacillales–Chitinophagales	
B10.SB3A–Subgroup.2	Bacillales–Sphingomonadales	
B10.SB3A–X24.Nov	Bacteroidales–Candidatus.Moranbacteria	
B12.WMSP1–CCD24	Bacteroidales–Defluviicoccales	
B12.WMSP1–Catenulisporales	Bacteroidales–Desulfobaccales	
B12.WMSP1–Ktedonobacterales	Bacteroidales–Desulfobulbales	
B12.WMSP1–Microtrichales	Bacteroidales–OPB41	
B12.WMSP1–Steroidobacterales	Bacteroidales–SJA.15	
B12.WMSP1–Subgroup.17	Bacteroidales–Spirochaetales	
B12.WMSP1–WD260	Bacteroidales–Syntrophobacterales	
Bacillales–Gemmatimonadales	Bryobacterales–C0119	
Blastocatellales–Subgroup.7	Bryobacterales–Chthonomonadales	
Blastocatellales–Thermoanaerobaculales	C0119–Candidatus.Adlerbacteria	
Bryobacterales–Candidatus.Liptonbacteria	C0119–Chthonomonadales	
Bryobacterales–Elsterales	C0119–Elsterales	
Bryobacterales–Gammaproteobacteria.Incertae.Sedis	C0119–Gammaproteobacteria.Incertae.Sedis	
Bryobacterales–Gemmatimonadales	C0119–Ktedonobacterales	
Burkholderiales–Caulobacterales	C0119–Micropepsales	
Burkholderiales–Haliangiales	C0119–PAUC26f	
Burkholderiales–Sphingobacteriales	C0119–Sphingomonadales	
C0119–Gemmatimonadales	Candidatus.Adlerbacteria–Desulfitobacteriales	
C0119–PLTA13	Candidatus.Adlerbacteria–Kryptoniales	
C0119–Solibacterales	Candidatus.Adlerbacteria–PAUC26f	
CCD24–Caldilineales	Candidatus.Moranbacteria–Desulfobaccales	
CCD24–Catenulisporales	Candidatus.Moranbacteria–Desulfobulbales	
CCD24–RBG.13.54.9	Candidatus.Moranbacteria–Ignavibacteriales	
CCD24–Rokubacteriales	Candidatus.Moranbacteria–Ktedonobacterales	
CCD24–Steroidobacterales	Candidatus.Moranbacteria–Syntrophobacterales	
CCD24–Subgroup.17	Candidatus.Yanofskybacteria–Kryptoniales	
CCD24–Vicinamibacterales	Candidatus.Yanofskybacteria–PLTA13	
Caldilineales–Catenulisporales	Candidatus.Yanofskybacteria–Subgroup.17	
Caldilineales–Microtrichales	Chitinophagales–Chthoniobacterales	
Caldilineales–Propionibacteriales	Chitinophagales–Sphingobacteriales	
Candidatus.Jorgensenbacteria–Candidatus.Kaiserbacteria	Cytophagales–Polyangiales	
Candidatus.Jorgensenbacteria–Candidatus.Liptonbacteria	Defluviicoccales–Desulfitobacteriales	
Candidatus.Jorgensenbacteria–KF.JG30.C25	Defluviicoccales–Kryptoniales	
Catenulisporales–Elsterales	Defluviicoccales–PAUC26f	
Catenulisporales–Kapabacteriales	Defluviicoccales–PLTA13	
Catenulisporales–Ktedonobacterales	Defluviicoccales–S085	
Catenulisporales–WD260	Defluviicoccales–SJA.15	
Caulobacterales–Rhizobiales	Desulfitobacteriales–Desulfobaccales	
Caulobacterales–Sphingomonadales	Desulfitobacteriales–Kryptoniales	
Caulobacterales–Xanthomonadales	Desulfobaccales–Desulfobulbales	
Chitinophagales–Micrococcales	Desulfobaccales–Ignavibacteriales	
Chitinophagales–Rhizobiales	Desulfobaccales–Kryptoniales	
Chloroflexales–Kallotenuales	Desulfobaccales–SJA.15	
Chloroflexales–Propionibacteriales	Desulfobaccales–Syntrophales	

Continued on next page

Table S17 Continued from previous page

Clean Network Links	Disease Network Links	Common Links
Chloroflexales–Pyrinomonadales	Desulfobaccales–Syntrophobacterales	
Chloroflexales–RBG.13.54.9	Desulfobulbales–Kryptoniales	
Chthoniobacterales–Solibacterales	Desulfobulbales–SJA.15	
Chthonomonadales–Elsterales	Desulfobulbales–Syntrophales	
Chthonomonadales–PLTA13	Desulfobulbales–Syntrophobacterales	
Cyanobacteriales–Leptolyngbyales	Elsterales–Gemmatimonadales	
DS.100–X24.Nov	Elsterales–Ktedonobacterales	
Diplorickettsiales–Xanthomonadales	Elsterales–Micropepsales	
Elev.1554–Gammaproteobacteria.Incertae.Sedis	Elsterales–Reyranellales	
Elev.1554–Gemmatales	Frankiales–Gemmatimonadales	
Elev.1554–Subgroup.13	Gammaproteobacteria.Incertae.Sedis–Paenibacillales	
Elev.1554–WD260	Gemmatimonadales–Kallotenuales	
Elsterales–Gammaproteobacteria.Incertae.Sedis	Ignavibacteriales–JG36.TzT.191	
Elsterales–Subgroup.13	Ignavibacteriales–Kryptoniales	
FFCH16263–Nitrospirales	Ignavibacteriales–Ktedonobacterales	
FFCH16263–Subgroup.2	Ignavibacteriales–Syntrophobacterales	
FFCH16263–Vicinamibacterales	Kryptoniales–Syntrophobacterales	
Frankiales–Gaiellales	Ktedonobacterales–Rickettsiales	
Gammaproteobacteria.Incertae.Sedis–Xanthomonadales	Ktedonobacterales–Syntrophobacterales	
Gemmatales–Isosphaerales	Methylomirabilales–OPB41	
Gemmatimonadales–Paenibacillales	Methylomirabilales–SJA.15	
Haliangiales–Pedosphaerales	Methylomirabilales–Syntrophales	
IMCC26256–Subgroup.2	OPB41–Syntrophales	
Isosphaerales–Ktedonobacterales	OPB41–Syntrophobacterales	
KF.JG30.C25–Subgroup.13	PAUC26f–PLTA13	
Ktedonobacterales–Micropepsales	PAUC26f–SJA.15	
Ktedonobacterales–PAUC26f	PLTA13–Syntrophobacterales	
Ktedonobacterales–Subgroup.2	Paenibacillales–Rickettsiales	
Leptolyngbyales–Oxyphotobacteria.Incertae.Sedis	Paenibacillales–Sphingomonadales	
Micromonosporales–Propionibacteriales	Paenibacillales–Xanthomonadales	
Micromonosporales–Rhizobiales	Propionibacteriales–Thermomicrobiales	
Micropepsales–Subgroup.2	Rhodothermales–SAR202.clade	
Micropepsales–Xanthomonadales	SJA.15–Syntrophobacterales	
Microtrichales–Phycisphaerales	Sphingomonadales–Xanthomonadales	
Microtrichales–Rhodobacterales	Spirochaetales–Syntrophales	
Microtrichales–SBR1031		
Microtrichales–Thermoanaerobaculales		
Microtrichales–Vicinamibacterales		
Nannocystales–PLTA13		
Nannocystales–Steroidobacterales		
Nannocystales–WD260		
Nitrospirales–Rokubacteriales		
Nitrospirales–Vicinamibacterales		
Pedosphaerales–Thermomicrobiales		
Phycisphaerales–SBR1031		
Propionibacteriales–Rhodobacterales		
Pyrinomonadales–RBG.13.54.9		
Pyrinomonadales–Vicinamibacterales		
Pyrinomonadales–X24.Nov		
RBG.13.54.9–Rokubacteriales		
RBG.13.54.9–SBR1031		
RBG.13.54.9–Subgroup.7		
Reyranellales–Solibacterales		
Rhodobacterales–Verrucomicrobiales		
Rhodobacterales–WD260		
Rokubacteriales–Subgroup.17		
Rokubacteriales–X24.Nov		
SBR1031–Steroidobacterales		
SBR1031–Subgroup.17		
SBR1031–Thermoanaerobaculales		
Saccharimonadales–Xanthomonadales		
Sphingobacteriales–Xanthomonadales		
Steroidobacterales–Vicinamibacterales		
Subgroup.17–Vicinamibacterales		
Subgroup.17–X24.Nov		

Continued on next page

Table S17 Continued from previous page

**Clean Network Links**	**Disease Network Links**	**Common Links**
Subgroup.2–WD260<br>Subgroup.7–Thermoanaerobaculales<br>Vicinamibacterales–X24.Nov		

Table S18: Important OTUs identified using Multi Machine Learning (ML) methods at the **Phylum** level. Microbiome data were normalized using four different techniques: (1) Centered Log-Ratio (CLR), (2) the original dataset (no transformation), (3) Log transformation, and (4) Total Sum Scaling (TSS). Seven ML-based feature selection methods were applied to each normalized dataset to identify important OTUs. An OTU was selected if it was identified as important by at least five out of the seven ML methods. The table presents the OTUs identified as important for each normalization method, with separate columns for ML_Clr (CLR-normalized data), ML_Original (original dataset), ML_Log (log-transformed data), and ML_TSS (TSS-normalized data). The final ML_Intersection column lists the OTUs that were consistently selected as important across all normalization methods, highlighting robust microbial taxa that are independent of the normalization approach.

ML_Clr	ML_Original	ML_Log	ML_TSS	ML_Intersection
Firmicutes	Armatimonadota	Firmicutes	Firmicutes	Firmicutes
Cyanobacteria	Firmicutes	Armatimonadota	Cyanobacteria	Cyanobacteria
Methylomirabilota	Deinococcota	Cyanobacteria	Patescibacteria	Armatimonadota
Armatimonadota	Cyanobacteria	Deinococcota	Armatimonadota	NB1.j
Deinococcota	NB1.j	Verrucomicrobiota	Bacteroidota	
WPS.2	Verrucomicrobiota	Spirochaetota	Spirochaetota	
Acidobacteriota	Patescibacteria	NB1.j	Methylomirabilota	
MBNT15	Methylomirabilota	Bacteroidota	Acidobacteriota	
NB1.j		WPS.2	Nitrospirota	
Bacteroidota			Desulfobacterota	
Verrucomicrobiota			NB1.j	

Table S19: Important OTUs identified using Multi Machine Learning (ML) methods at the **Class** level. Microbiome data were normalized using four different techniques: (1) Centered Log-Ratio (CLR), (2) the original dataset (no transformation), (3) Log transformation, and (4) Total Sum Scaling (TSS). Seven ML-based feature selection methods were applied to each normalized dataset to identify important OTUs. An OTU was selected if it was identified as important by at least five out of the seven ML methods. The table presents the OTUs identified as important for each normalization method, with separate columns for ML_Clr (CLR-normalized data), ML_Original (original dataset), ML_Log (log-transformed data), and ML_TSS (TSS-normalized data). The final ML_Intersection column lists the OTUs that were consistently selected as important across all normalization methods, highlighting robust microbial taxa that are independent of the normalization approach.

ML_Clr	ML_Original	ML_Log	ML_TSS	ML_Intersection
Bacilli	Bacilli	Bacilli	Vicinamibacteria	Bacilli
Ktedonobacteria	Latescibacteria	Ignavibacteria	Saccharimonadia	Ktedonobacteria
Cyanobacteriia	Saccharimonadia	Verrucomicrobiae	Gemmatimonadetes	Cyanobacteriia
Saccharimonadia	Ktedonobacteria	Deinococci	Gitt.GS.136	Saccharimonadia
Planctomycetes	Spirochaetia	Latescibacteria	Oligoflexia	Planctomycetes
Lineage.IIa	Planctomycetes	Saccharimonadia	Ktedonobacteria	Ignavibacteria
Vampirivibrionia	Kryptonia	Ktedonobacteria	Anaerolineae	Dehalococcoidia
Verrucomicrobiae	Verrucomicrobiae	OLB14	Bacilli	Anaerolineae
Ignavibacteria	Deinococci	Kryptonia	Acidimicrobiia	MB.A2.108
Dehalococcoidia	Ignavibacteria	MB.A2.108	Planctomycetes	Chthonomonadetes
Thermoplasmata	OLB14	Cyanobacteriia	Nitrospiria	Kryptonia
Deinococci	Cyanobacteriia	Planctomycetes	Methylomirabilia	
Anaerolineae	Dehalococcoidia	AD3	Ignavibacteria	
Pla4.lineage	Methylomirabilia	Gitt.GS.136	MB.A2.108	
MB.A2.108	Chthonomonadetes	Dehalococcoidia	Acidobacteriae	
Methylomirabilia	MB.A2.108	Anaerolineae	TK10	
Chthonomonadetes	Gitt.GS.136	Armatimonadia	Bacteroidia	
OLB14	Vicinamibacteria	Chthonomonadetes	Polyangia	
Bacteroidia	Anaerolineae	Oligoflexia	Thermoleophilia	
Oligoflexia	Vampirivibrionia	Clostridia	KD4.96	
Kryptonia	Holophagae	Spirochaetia	Cyanobacteriia	
TK10			Chthonomonadetes	
			Dehalococcoidia	
			Kryptonia	

Table S20: Important OTUs identified using Multi Machine Learning (ML) methods at the **Order** level. Microbiome data were normalized using four different techniques: (1) Centered Log-Ratio (CLR), (2) the original dataset (no transformation), (3) Log transformation, and (4) Total Sum Scaling (TSS). Seven ML-based feature selection methods were applied to each normalized dataset to identify important OTUs. An OTU was selected if it was identified as important by at least five out of the seven ML methods. The table presents the OTUs identified as important for each normalization method, with separate columns for ML_Clr (CLR-normalized data), ML_Original (original dataset), ML_Log (log-transformed data), and ML_TSS (TSS-normalized data). The final ML_Intersection column lists the OTUs that were consistently selected as important across all normalization methods, highlighting robust microbial taxa that are independent of the normalization approach.

ML_Clr	ML_Original	ML_Log	ML_TSS	ML_Intersection
Saccharimonadales	Anaerolineales	Saccharimonadales	Sphingomonadales	Saccharimonadales
Bacillales	Acetobacterales	Bacillales	Saccharimonadales	Bacillales
C0119	Chloroplast	Subgroup.2	SBR1031	C0119
Isosphaerales	PAUC26f	Solibacterales	Kryptoniales	Subgroup.2
Subgroup.2	Subgroup.17	Micropepsales	Vicinamibacterales	Xanthomonadales
Blastocatellales	Bacteroidales	Anaerolineales	Anaerolineales	Acidobacteriales
Xanthomonadales	Alicyclobacillales	Latescibacterales	C0119	Chloroplast
Acidobacteriales	Micropepsales	Frankiales	Bryobacterales	Alicyclobacillales
Chloroplast	Bacillales	Gaiellales	Micropepsales	Paenibacillales
Alicyclobacillales	Sphingomonadales	Alicyclobacillales	Chloroplast	Acetobacterales
Paenibacillales	Defluviicoccales	Bacteroidales	Subgroup.2	Pseudomonadales
Acetobacterales	Frankiales	B10.SB3A	Gaiellales	Anaerolineales
Pseudomonadales	Saccharimonadales	Acetobacterales	PLTA13	Elsterales
Anaerolineales	Caulobacterales	Chloroplast	Bacillales	Bacteroidales
Elsterales	Acidobacteriales	Defluviicoccales	Microtrichales	Ktedonobacterales
Oligoflexales	Pseudomonadales	Propionibacteriales	Candidatus.Levybacteria	Sphingomonadales
Bacteroidales	Bryobacterales	Acidobacteriales	Pseudomonadales	Kineosporiales
Deinococcales	C0119	Sphingomonadales	Rokubacteriales	SBR1031
Ktedonobacterales	Gaiellales	Caulobacterales	Acetobacterales	Rokubacteriales
Sphingomonadales	B10.SB3A	B12.WMSP1	Clostridiales	Frankiales
Chloroflexales	Microtrichales	SBR1031	PAUC26f	Micropepsales
Rhodobacterales	Chthoniobacterales	Chthoniobacterales	Elsterales	Gaiellales
Candidatus.Yanofskybacteria	SBR1031	Deinococcales	Frankiales	PLTA13
KF.JG30.C25	Kineosporiales	Pseudomonadales	B10.SB3A	Defluviicoccales
Kineosporiales	Ktedonobacterales	Obscuribacterales	Kineosporiales	Obscuribacterales
SBR1031	Subgroup.2	Rokubacteriales	Ktedonobacterales	
Rokubacteriales	Latescibacterales	Subgroup.17	Subgroup.17	
Frankiales	Chitinophagales	Lineage.IV	Lineage.IV	
Rickettsiales	B12.WMSP1	PAUC26f	Micromonosporales	
Micropepsales	Blastocatellales	Spirochaetales	Gammaproteobacteria.Incertae	
Enterobacterales	Xanthomonadales	Chitinophagales	Blastocatellales	
Micrococcales	Syntrophobacterales	Kineosporiales	Cytophagales	
Gaiellales	Sphingobacteriales	Isosphaerales	Obscuribacterales	
PLTA13	SJA.15	Paenibacillales	Deinococcales	
Solibacterales	Elsterales	Xanthomonadales	Chthoniobacterales	
CCD24	Pseudonocardiales	C0119	Chthonomonadales	
Defluviicoccales	Clostridiales	Ktedonobacterales	Acidobacteriales	
Desulfitobacteriales	Candidatus.Yanofskybacteria	Micromonosporales	Pseudonocardiales	
S085	Kryptoniales	Kryptoniales	Actinomarinales	
Obscuribacterales	Chthonomonadales	Chthonomonadales	Rickettsiales	
	SJA.28	Thermoanaerobaculales	Paenibacillales	
	Paenibacillales	PLTA13	Alicyclobacillales	
	Propionibacteriales	Bryobacterales	Xanthomonadales	
	S085	Rickettsiales	SJA.28	
	PLTA13	Elsterales	Chitinophagales	
	Isosphaerales		Ardenticatenales	
	Obscuribacterales		Bacteroidales	
	Rokubacteriales		Gemmatimonadales	
	Solibacterales		Nitrospirales	
			Rhizobiales	
			Defluviicoccales	
			CCD24	
			Solirubrobacterales	
			S085	

Table S21: Important OTUs identified as key features in response to pitted scab at the **Phylum level using Strategy 1: Differential Centrality Analysis.** First, microbiome networks were constructed separately for 'scab-infected tubers' and 'clean tubers' using four inference methods: `SE_glasso`, `SPRING`, `SPARCC`, and `CMIMN`. Then, centrality metrics (Degree, Betweenness, Closeness, Eigenvector, and PageRank) were calculated for both networks, and the differences in centrality values between diseased and healthy conditions were computed for each method. OTUs ranked in the top 20% based on these centrality differences across all four methods were selected as important. The table presents OTUs that were consistently identified as important across all four inference methods. The Features column indicates the centrality measure that determined the significance of each OTU. These OTUs represent microbial taxa whose network connectivity consistently exhibited significant differences between healthy and diseased conditions across all four methods.

Important OTUs	Topological Features
Bacteroidota	Betweenness
WPS.2	Betweenness
Proteobacteria	Closeness

Table S22: Important OTUs identified as key features in response to pitted scab at the **Class level using Strategy 1: Differential Centrality Analysis.** First, microbiome networks were constructed separately for 'scab-infected tubers' and 'clean tubers' using four inference methods: `SE_glasso`, `SPRING`, `SPARCC`, and `CMIMN`. Then, centrality metrics (Degree, Betweenness, Closeness, Eigenvector, and PageRank) were calculated for both networks, and the differences in centrality values between diseased and healthy conditions were computed for each method. OTUs ranked in the top 20% based on these centrality differences across all four methods were selected as important. The table presents OTUs that were consistently identified as important across all four inference methods. The Features column indicates the centrality measure that determined the significance of each OTU. These OTUs represent microbial taxa whose network connectivity consistently exhibited significant differences between healthy and diseased conditions across all four methods.

Important OTUs	Topological Features
Desulfitobacteriia	degree, Eigenvector Centrality, page_rank
Actinobacteria, AD3	betweenness, closeness
Syntrophobacteria	Eigenvector Centrality

Table S23: Important OTUs identified as key features in response to pitted scab at the **Order level using Strategy 1: Differential Centrality Analysis.** First, microbiome networks were constructed separately for 'scab-infected tubers' and 'clean tubers' using four inference methods: `SE_glasso`, `SPRING`, `SPARCC`, and `CMIMN`. Then, centrality metrics (Degree, Betweenness, Closeness, Eigenvector, and PageRank) were calculated for both networks, and the differences in centrality values between diseased and healthy conditions were computed for each method. OTUs ranked in the top 20% based on these centrality differences across all four methods were selected as important. The table presents OTUs that were consistently identified as important across all four inference methods. The Features column indicates the centrality measure that determined the significance of each OTU. These OTUs represent microbial taxa whose network connectivity consistently exhibited significant differences between healthy and diseased conditions across all four methods.

Important OTUs	Topological Features
C0119	Degree, Betweenness, Closeness, Eigenvector Centrality, Page Rank
Defluviicoccales	Closeness, Eigenvector Centrality
Bacteroidales	Closeness, Eigenvector Centrality
Kryptoniales	Eigenvector Centrality
B12.WMSP1	Eigenvector Centrality
Desulfitobacteriales	Page Rank

Table S24: Selection of key Operational Taxonomic Units (OTUs) at the **Phylum** level in microbiome networks constructed separately for 'clean tubers' (left panel) and 'scab-infected tubers' (right panel) using network-based feature selection (Strategy 2: Composite Scoring Approach). Steps for Identifying These OTUs: 1- Network Construction: Microbiome networks were separately built for clean tubers and scab-infected tubers using four inference methods: `SE_glasso`, `SPRING`, `SPARCC`, and `CMIMN`. 2- Weighted Scoring Within Each Method: A weighted score was assigned to each OTU within each method based on multiple centrality metrics (Degree, Betweenness, Closeness, Eigenvector, and PageRank). Selection of Important OTUs: The top 20% of OTUs with the highest Score 1 were selected within each individual method. Table Column Explanations: First column in each panel: OTUs selected by Strategy 2 for each specific network inference method (i.e., these OTUs are among the top 20% highest-scoring OTUs for that method). Next four columns (CLR, Original, Log, TSS): Overlap between Strategy 2-selected OTUs and those identified by ML-based feature selection under different normalization approaches. A 1 in a column means that the ML method also identified the OTU as important under that normalization method. A 0 in a column means that the OTU was not selected by the ML method under that normalization method. Note: This table presents the weighted score for each OTU within each inference method. Unlike later steps in Strategy 2, this table does not include the combined score across all methods.

**'clean tubers' network**					**'scab-infected tubers' network**				
`CMIMN`	clr	original	log	TSS	`CMIMN`	clr	original	log	TSS
Methylomirabilota	1	1	0	1	Nitrospirota	0	0	0	1
Myxococcota	0	0	0	0	Desulfobacterota	0	0	0	1
Nitrospirota	0	0	0	1	Myxococcota	0	0	0	0
Desulfobacterota	0	0	0	1	Bacteroidota	1	0	1	1
MBNT15	1	0	0	0	NB1.j	1	1	1	1
Armatimonadota	1	1	1	1	Patescibacteria	0	1	0	1
Proteobacteria	0	0	0	0	Proteobacteria	0	0	0	0
WPS.2	1	0	1	0	Acidobacteriota	1	0	0	1
`SPARCC`					`SPARCC`				
WPS.2	1	0	1	0	Firmicutes	1	1	1	1
Methylomirabilota	1	1	0	1	Desulfobacterota	0	0	0	1
Acidobacteriota	1	0	0	1	Gemmatimonadota	0	0	0	0
Proteobacteria	0	0	0	0	Acidobacteriota	1	0	0	1
Patescibacteria	0	1	0	1	Proteobacteria	0	0	0	0
Gemmatimonadota	0	0	0	0	Verrucomicrobiota	1	1	1	0
Actinobacteriota	0	0	0	0	Bacteroidota	1	0	1	1
Planctomycetota	0	0	0	0	Cyanobacteria	1	1	1	1
`SE_glasso`					`SE_glasso`				
WPS.2	1	0	1	0	Proteobacteria	0	0	0	0
Acidobacteriota	1	0	0	1	MBNT15	1	0	0	0
Proteobacteria	0	0	0	0	Myxococcota	0	0	0	0
Chloroflexi	0	0	0	0	Bacteroidota	1	0	1	1
Patescibacteria	0	1	0	1	NB1.j	1	1	1	1
Planctomycetota	0	0	0	0	Desulfobacterota	0	0	0	1
Gemmatimonadota	0	0	0	0	Firmicutes	1	1	1	1
Armatimonadota	1	1	1	1	Actinobacteriota	0	0	0	0
`SPRING`					`SPRING`				
WPS.2	1	0	1	0	Proteobacteria	0	0	0	0
NB1.j	1	1	1	1	Chloroflexi	0	0	0	0
Patescibacteria	0	1	0	1	Nitrospirota	0	0	0	1
Actinobacteriota	0	0	0	0	Bacteroidota	1	0	1	1
Methylomirabilota	1	1	0	1	Firmicutes	1	1	1	1
Proteobacteria	0	0	0	0	NB1.j	1	1	1	1
GAL15	0	0	0	0	RCP2.54	0	0	0	0
MBNT15	1	0	0	0	Acidobacteriota	1	0	0	1

Table S25: Selection of key Operational Taxonomic Units (OTUs) at the **Class** level in microbiome networks constructed separately for 'clean tubers' (left panel) and 'scab-infected tubers' (right panel) using network-based feature selection (Strategy 2: Composite Scoring Approach). Steps for Identifying These OTUs: 1- Network Construction: Microbiome networks were separately built for clean tubers and scab-infected tubers using four inference methods: `SE_glasso`, `SPRING`, `SPARCC`, and `CMIMN`. 2- Weighted Scoring Within Each Method: A weighted score was assigned to each OTU within each method based on multiple centrality metrics (Degree, Betweenness, Closeness, Eigenvector, and PageRank). Selection of Important OTUs: The top 20% of OTUs with the highest Score 1 were selected within each individual method. Table Column Explanations: First column in each panel: OTUs selected by Strategy 2 for each specific network inference method (i.e., these OTUs are among the top 20% highest-scoring OTUs for that method). Next four columns (CLR, Original, Log, TSS): Overlap between Strategy 2-selected OTUs and those identified by ML-based feature selection under different normalization approaches. A 1 in a column means that the ML method also identified the OTU as important under that normalization method. A 0 in a column means that the OTU was not selected by the ML method under that normalization method. Note: This table presents the weighted score for each OTU within each inference method. Unlike later steps in Strategy 2, this table does not include the combined score across all methods.

**clean tuber Net**	clr	original	log	TSS	**scab-infected tubers Net**	clr	original	log	TSS
**CMIMN**									
Ktedonobacteria	1	1	1	1	Ignavibacteria	1	1	1	1
Ignavibacteria	1	1	1	1	Parcubacteria	0	0	0	0
OM190	0	0	0	0	Anaerolineae	1	1	1	1
Acidimicrobiia	0	0	0	1	Vicinamibacteria	0	1	0	1
Vicinamibacteria	0	1	0	1	Kryptonia	1	1	1	1
Actinobacteria	0	0	0	0	Acidimicrobiia	0	0	0	1
Desulfobaccia	0	0	0	0	Gammaproteobacteria	0	0	0	0
Gammaproteobacteria	0	0	0	0	Alphaproteobacteria	0	0	0	0
Alphaproteobacteria	0	0	0	0	AD3	0	0	1	0
Blastocatellia	0	0	0	0	Acidobacteriae	0	0	0	1
Gitt.GS.136	0	1	1	1	Nitrospiria	0	0	0	1
Parcubacteria	0	0	0	0	Microgenomatia	0	0	0	0
Acidobacteriae	0	0	0	1	Myxococcia	0	0	0	0
Polyangia	0	0	0	1	BD2.11.terrestrial.group	0	0	0	0
Chloroflexia	0	0	0	0	Thermoleophilia	0	0	0	1
KD4.96	0	0	0	1	Gitt.GS.136	0	1	1	1
Thermoleophilia	0	0	0	1	Blastocatellia	0	0	0	0
MB.A2.108	1	1	1	1	Holophagae	0	1	0	0
Babeliae	0	0	0	0	KD4.96	0	0	0	1
Longimicrobia	0	0	0	0	Bacilli	1	1	1	1
**SPARCC**									
Vicinamibacteria	0	1	0	1	Actinobacteria	0	0	0	0
Anaerolineae	1	1	1	1	Bacilli	1	1	1	1
OLB14	1	1	1	0	AD3	0	0	1	0
Actinobacteria	0	0	0	0	Acidobacteriae	0	0	0	1
Ktedonobacteria	1	1	1	1	Anaerolineae	1	1	1	1
Methylomirabilia	1	1	0	1	Blastocatellia	0	0	0	0
Acidobacteriae	0	0	0	1	Chloroflexia	0	0	0	0
Bacteroidia	1	0	0	1	Thermoleophilia	0	0	0	1
Saccharimonadia	1	1	1	1	Ignavibacteria	1	1	1	1
Chlamydiae	0	0	0	0	Ktedonobacteria	1	1	1	1
Alphaproteobacteria	0	0	0	0	Nitrospiria	0	0	0	1

Continued on next page

Planctomycetes	1	1	1	1	Parcubacteria	0	0	0	0
Gammaproteobacteria	0	0	0	0	Alphaproteobacteria	0	0	0	0
MB.A2.108	1	1	1	1	Rhodothermia	0	0	0	0
Gemmatimonadetes	0	0	0	1	Kryptonia	1	1	1	1
Gitt.GS.136	0	1	1	1	Gemmatimonadetes	0	0	0	1
AD3	0	0	1	0	Syntrophobacteria	0	0	0	0
Subgroup.25	0	0	0	0	Thermodesulfovibrionia	0	0	0	0
Blastocatellia	0	0	0	0	Polyangia	0	0	0	1
Thermoanaerobaculia	0	0	0	0	Gammaproteobacteria	0	0	0	0
**SE_glasso**									
Ktedonobacteria	1	1	1	1	AD3	0	0	1	0
Syntrophobacteria	0	0	0	0	Ktedonobacteria	1	1	1	1
Anaerolineae	1	1	1	1	Bacilli	1	1	1	1
Acidobacteriae	0	0	0	1	Desulfobaccia	0	0	0	0
Desulfobaccia	0	0	0	0	Gammaproteobacteria	0	0	0	0
AD3	0	0	1	0	Desulfobulbia	0	0	0	0
Vicinamibacteria	0	1	0	1	Syntrophobacteria	0	0	0	0
Actinobacteria	0	0	0	0	Thermodesulfovibrionia	0	0	0	0
Thermoleophilia	0	0	0	1	Alphaproteobacteria	0	0	0	0
Planctomycetes	1	1	1	1	Ignavibacteria	1	1	1	1
Methylomirabilia	1	1	0	1	Kryptonia	1	1	1	1
MB.A2.108	1	1	1	1	Actinobacteria	0	0	0	0
Gemmatimonadetes	0	0	0	1	Blastocatellia	0	0	0	0
Alphaproteobacteria	0	0	0	0	Anaerolineae	1	1	1	1
Gammaproteobacteria	0	0	0	0	Dehalococcoidia	1	1	1	1
Latescibacteria	0	1	1	0	Verrucomicrobiae	1	1	1	0
Subgroup.25	0	0	0	0	Acidobacteriae	0	0	0	1
Gitt.GS.136	0	1	1	1	TK10	1	0	0	1
Saccharimonadia	1	1	1	1	Syntrophia	0	0	0	0
Desulfobulbia	0	0	0	0	Gemmatimonadetes	0	0	0	1
**SPRING**									
Actinobacteria	0	0	0	0	AD3	0	0	1	0
Blastocatellia	0	0	0	0	Syntrophobacteria	0	0	0	0
Vicinamibacteria	0	1	0	1	Bacteroidia	1	0	0	1
Acidimicrobiia	0	0	0	1	Rhodothermia	0	0	0	0
Alphaproteobacteria	0	0	0	0	Polyangia	0	0	0	1
Acidobacteriae	0	0	0	1	Nitrospiria	0	0	0	1
Parcubacteria	0	0	0	0	Alphaproteobacteria	0	0	0	0
Polyangia	0	0	0	1	Bdellovibrionia	0	0	0	0
Gammaproteobacteria	0	0	0	0	Cyanobacteriia	1	1	1	1
Rhodothermia	0	0	0	0	Acidimicrobiia	0	0	0	1
Longimicrobia	0	0	0	0	Microgenomatia	0	0	0	0
AKAU4049	0	0	0	0	Kazania	0	0	0	0
Anaerolineae	1	1	1	1	Parcubacteria	0	0	0	0
Ktedonobacteria	1	1	1	1	Gammaproteobacteria	0	0	0	0
Kazania	0	0	0	0	Thermoleophilia	0	0	0	1
Gracilibacteria	0	0	0	0	Blastocatellia	0	0	0	0

Continued on next page

Gemmatimonadetes	0	0	0	1	OM190	0	0	0	0
Thermoplasmata	1	0	0	0	Desulfitobacteriia	0	0	0	0
Verrucomicrobiae	1	1	1	0	Anaerolineae	1	1	1	1
Desulfobulbia	0	0	0	0	Bacilli	1	1	1	1

Table S26: Selection of key Operational Taxonomic Units (OTUs) at the **Order** level in microbiome networks constructed separately for 'clean tubers' (left panel) and 'scab-infected tubers' (right panel) using network-based feature selection (Strategy 2: Composite Scoring Approach). Steps for Identifying These OTUs: 1- Network Construction: Microbiome networks were separately built for clean tubers and scab-infected tubers using four inference methods: `SE_glasso`, `SPRING`, `SPARCC`, and `CMIMN`. 2- Weighted Scoring Within Each Method: A weighted score was assigned to each OTU within each method based on multiple centrality metrics (Degree, Betweenness, Closeness, Eigenvector, and PageRank). Selection of Important OTUs: The top 20% of OTUs with the highest Score 1 were selected within each individual method. Table Column Explanations: First column in each panel: OTUs selected by Strategy 2 for each specific network inference method (i.e., these OTUs are among the top 20% highest-scoring OTUs for that method). Next four columns (CLR, Original, Log, TSS): Overlap between Strategy 2-selected OTUs and those identified by ML-based feature selection under different normalization approaches. A 1 in a column means that the ML method also identified the OTU as important under that normalization method. A 0 in a column means that the OTU was not selected by the ML method under that normalization method. Note: This table presents the weighted score for each OTU within each inference method. Unlike later steps in Strategy 2, this table does not include the combined score across all methods.

**clean tuber Net**	clr	original	log	TSS	**scab-infected tubers Net**	clr	original	log	TSS
**CMIMN**									
Ktedonobacterales	1	1	1	1	Ktedonobacterales	1	1	1	1
C0119	1	1	1	1	Elev.1554	0	0	0	0
Acidobacteriales	1	1	1	1	Microtrichales	0	1	0	1
Rhizobiales	0	0	0	1	Bryobacterales	0	1	1	1
Bacillales	1	1	1	1	C0119	1	1	1	1
Propionibacteriales	0	1	1	0	Acetobacterales	1	1	1	1
Micropepsales	1	1	1	1	Haliangiales	0	0	0	0
PLTA13	1	1	1	1	Pedosphaerales	0	0	0	0
Chitinophagales	0	1	1	1	Rickettsiales	1	0	1	1
Actinomarinales	0	0	0	1	Rhizobiales	0	0	0	1
Subgroup.17	0	1	1	1	Phycisphaerales	0	0	0	0
Chloroplast	1	1	1	1	PLTA13	1	1	1	1
Bryobacterales	0	1	1	1	Chloroplast	1	1	1	1
Rhodobacterales	1	0	0	0	Subgroup.17	0	1	1	1
B12.WMSP1	0	1	1	0	Subgroup.2	1	1	1	1
Elev.1554	0	0	0	0	Sphingobacteriales	0	1	0	0
Chloroflexales	1	0	0	0	Reyranellales	0	0	0	0
Acetobacterales	1	1	1	1	Actinomarinales	0	0	0	1
Desulfobaccales	0	0	0	0	Thermoactinomycetales	0	0	0	0
Elsterales	1	1	1	1	S085	1	1	0	1
Gaiellales	1	1	1	1	Erysipelotrichales	0	0	0	0
B10.SB3A	0	1	1	1	SBR1031	1	1	1	1
Microtrichales	0	1	0	1	Gaiellales	1	1	1	1
Gemmatimonadales	0	0	0	1	Myxococcales	0	0	0	0
Vicinamibacterales	0	0	0	1	Kryptoniales	0	1	1	1
Micrococcales	1	0	0	0	Opitutales	0	0	0	0

Continued on next page

Table S26 – continued from previous page

**clean tuber Net**	clr	original	log	TSS	**scab-infected tubers Net**	clr	original	log	TSS
Frankiales	1	1	1	1	CCD24	1	0	0	1
Subgroup.2	1	1	1	1	Solirubrobacterales	0	0	0	1
Thermoanaerobaculales	0	0	1	0	Blastocatellales	1	1	0	1
Anaerolineales	1	1	1	1	Saccharimonadales	1	1	1	1
Bacteroidales	1	1	1	1	Micromonosporales	0	0	1	1
CCD24	1	0	0	1	IMCC26256	0	0	0	0
Verrucomicrobiales	0	0	0	0	Ardenticatenales	0	0	0	1
Kryptoniales	0	1	1	1	Desulfitobacteriales	1	0	0	0
Sphingobacteriales	0	1	0	0	Candidatus.Yanofskybacteria	1	1	0	0
Ignavibacteriales	0	0	0	0	Catenulisporales	0	0	0	0
Burkholderiales	0	0	0	0	PAUC26f	0	1	1	1
Babeliales	0	0	0	0	Streptomycetales	0	0	0	0
**SPARCC**									
C0119	1	1	1	1	Ktedonobacterales	1	1	1	1
Microtrichales	0	1	0	1	Bacteroidales	1	1	1	1
Acidobacteriales	1	1	1	1	Gemmatimonadales	0	0	0	1
Vicinamibacterales	0	0	0	1	Bryobacterales	0	1	1	1
Elsterales	1	1	1	1	Microtrichales	0	1	0	1
Chloroplast	1	1	1	1	Subgroup.17	0	1	1	1
Ktedonobacterales	1	1	1	1	Thermomicrobiales	0	0	0	0
Bryobacterales	0	1	1	1	Blastocatellales	1	1	0	1
Xanthomonadales	1	1	1	1	Sphingomonadales	1	1	1	1
Leptolyngbyales	0	0	0	0	Micrococcales	1	0	0	0
Elev.1554	0	0	0	0	Elsterales	1	1	1	1
Micropepsales	1	1	1	1	Actinomarinales	0	0	0	1
Acetobacterales	1	1	1	1	Ignavibacteriales	0	0	0	0
Chloroflexales	1	0	0	0	Reyranellales	0	0	0	0
B12.WMSP1	0	1	1	0	Anaerolineales	1	1	1	1
Chitinophagales	0	1	1	1	C0119	1	1	1	1
Sphingomonadales	1	1	1	1	Solibacterales	1	1	1	0
B10.SB3A	0	1	1	1	Streptomycetales	0	0	0	0
Frankiales	1	1	1	1	SJA.15	0	1	0	0
Solibacterales	1	1	1	0	Frankiales	1	1	1	1
Gaiellales	1	1	1	1	Chloroflexales	1	0	0	0
X24.Nov	0	0	0	0	Defluviicoccales	1	1	1	1
SBR1031	1	1	1	1	Candidatus.Yanofskybacteria	1	1	0	0
Subgroup.2	1	1	1	1	Chitinophagales	0	1	1	1
Propionibacteriales	0	1	1	0	Bacillales	1	1	1	1
Paenibacillales	1	1	1	1	Erysipelotrichales	0	0	0	0
Nitrospirales	0	0	0	1	Kryptoniales	0	1	1	1
Saccharimonadales	1	1	1	1	Acidobacteriales	1	1	1	1
Sphingobacteriales	0	1	0	0	Streptosporangiales	0	0	0	0
Bacillales	1	1	1	1	Azospirillales	0	0	0	0
CCD24	1	0	0	1	Acetobacterales	1	1	1	1
Subgroup.17	0	1	1	1	SBR1031	1	1	1	1

Continued on next page

Table S26 – continued from previous page

**clean tuber Net**	clr	original	log	TSS	**scab-infected tubers Net**	clr	original	log	TSS
Rhizobiales	0	0	0	1	Syntrophobacterales	0	1	0	0
Nannocystales	0	0	0	0	Gammaproteobacteria.Incertae	0	0	0	1
Catenulisporales	0	0	0	0	CCD24	1	0	0	1
Rokubacteriales	1	1	1	1	Desulfobaccales	0	0	0	0
Caldilineales	0	0	0	0	Xanthomonadales	1	1	1	1
Pyrinomonadales	0	0	0	0	Sphingobacteriales	0	1	0	0
**SE_glasso**									
Ktedonobacterales	1	1	1	1	Sphingomonadales	1	1	1	1
Acetobacterales	1	1	1	1	Chitinophagales	0	1	1	1
Elsterales	1	1	1	1	Ktedonobacterales	1	1	1	1
Microtrichales	0	1	0	1	Elev.1554	0	0	0	0
Xanthomonadales	1	1	1	1	Ignavibacteriales	0	0	0	0
Vicinamibacterales	0	0	0	1	Bacteroidales	1	1	1	1
Desulfobaccales	0	0	0	0	Pedosphaerales	0	0	0	0
Ignavibacteriales	0	0	0	0	Subgroup.17	0	1	1	1
Micropepsales	1	1	1	1	C0119	1	1	1	1
Acidobacteriales	1	1	1	1	Defluviicoccales	1	1	1	1
SBR1031	1	1	1	1	PLTA13	1	1	1	1
C0119	1	1	1	1	Elsterales	1	1	1	1
SJA.15	0	1	0	0	Burkholderiales	0	0	0	0
Bryobacterales	0	1	1	1	Syntrophobacterales	0	1	0	0
Sphingomonadales	1	1	1	1	SJA.15	0	1	0	0
Subgroup.2	1	1	1	1	Desulfobulbales	0	0	0	0
B10.SB3A	0	1	1	1	Gemmatimonadales	0	0	0	1
PAUC26f	0	1	1	1	Anaerolineales	1	1	1	1
Elev.1554	0	0	0	0	Actinomarinales	0	0	0	1
Rhizobiales	0	0	0	1	PAUC26f	0	1	1	1
Caldilineales	0	0	0	0	Rhodothermales	0	0	0	0
B12.WMSP1	0	1	1	0	Frankiales	1	1	1	1
Propionibacteriales	0	1	1	0	Candidatus.Yanofskybacteria	1	1	0	0
Solibacterales	1	1	1	0	Desulfobaccales	0	0	0	0
Caulobacterales	0	1	1	0	Paenibacillales	1	1	1	1
Actinomarinales	0	0	0	1	Kryptoniales	0	1	1	1
Chloroflexales	1	0	0	0	Bryobacterales	0	1	1	1
Subgroup.17	0	1	1	1	Acidobacteriales	1	1	1	1
Candidatus.Jorgensenbacteria	0	0	0	0	Methylomirabilales	0	0	0	0
Rhodobacterales	1	0	0	0	Armatimonadales	0	0	0	0
Chitinophagales	0	1	1	1	Reyranellales	0	0	0	0
Frankiales	1	1	1	1	Syntrophales	0	0	0	0
Syntrophobacterales	0	1	0	0	Acetobacterales	1	1	1	1
Methylomirabilales	0	0	0	0	Bacillales	1	1	1	1
Gemmatimonadales	0	0	0	1	Micrococcales	1	0	0	0
Aminicenantales	0	0	0	0	Microtrichales	0	1	0	1
Desulfobulbales	0	0	0	0	AKIW659	0	0	0	0
Syntrophales	0	0	0	0	Ardenticatenales	0	0	0	1

Continued on next page

Table S26 – continued from previous page

**clean tuber Net**	clr	original	log	TSS	**scab-infected tubers Net**	clr	original	log	TSS
**SPRING**									
JG36.TzT.191	0	0	0	0	Erysipelotrichales	0	0	0	0
Paludibaculum	0	0	0	0	Opitutales	0	0	0	0
Caldilineales	0	0	0	0	Candidatus.Adlerbacteria	0	0	0	0
KF.JG30.C25	1	0	0	0	Brevibacillales	0	0	0	0
Haliangiales	0	0	0	0	Elev.16S.1166	0	0	0	0
Gammaproteobacteria.Incertae	0	0	0	1	Sphingobacteriales	0	1	0	0
JG36.GS.52	0	0	0	0	Cytophagales	0	0	0	1
Bacteroidales	1	1	1	1	Flavobacteriales	0	0	0	0
C0119	1	1	1	1	Enterobacterales	1	0	0	0
Rhodothermales	0	0	0	0	Haliangiales	0	0	0	0
Erysipelotrichales	0	0	0	0	Chloroplast	1	1	1	1
Chitinophagales	0	1	1	1	Xanthomonadales	1	1	1	1
Paenibacillales	1	1	1	1	WD260	0	0	0	0
PB19	0	0	0	0	KF.JG30.C25	1	0	0	0
SJA.15	0	1	0	0	Paracaedibacterales	0	0	0	0
SAR202.clade	0	0	0	0	Syntrophales	0	0	0	0
Chloroplast	1	1	1	1	Isosphaerales	1	1	1	0
Thermomicrobiales	0	0	0	0	PAUC26f	0	1	1	1
Sphingomonadales	1	1	1	1	Desulfitobacteriales	1	0	0	0
Rhodospirillales	0	0	0	0	Sphingomonadales	1	1	1	1
MSB.4B10	0	0	0	0	Steroidobacterales	0	0	0	0
PAUC26f	0	1	1	1	JG36.GS.52	0	0	0	0
SBR1031	1	1	1	1	Paludibaculum	0	0	0	0
Entomoplasmatales	0	0	0	0	PB19	0	0	0	0
S085	1	1	0	1	SJA.28	0	1	0	1
Isosphaerales	1	1	1	0	Saccharimonadales	1	1	1	1
Polyangiales	0	0	0	0	Obscuribacterales	1	1	1	1
Defluviicoccales	1	1	1	1	Rickettsiales	1	0	1	1
Elev.16S.1166	0	0	0	0	Fibrobacterales	0	0	0	0
Candidatus.Kaiserbacteria	0	0	0	0	Chitinophagales	0	1	1	1
Candidatus.Liptonbacteria	0	0	0	0	Pyrinomonadales	0	0	0	0
Xanthomonadales	1	1	1	1	Lactobacillales	0	0	0	0
FFCH16263	0	0	0	0	FCPU453	0	0	0	0
Subgroup.7	0	0	0	0	Ktedonobacterales	1	1	1	1
Rhodobacterales	1	0	0	0	Chthoniobacterales	0	1	1	1
Rhizobiales	0	0	0	1	Oxyphotobacteria.Incertae.Sedis	0	0	0	0
Pedosphaerales	0	0	0	0	Kineosporiales	1	1	1	1
Blastocatellales	1	1	0	1	Microtrichales	0	1	0	1

Table S27: Selection of Operational Taxonomic Units (OTUs) at the **Phylum** level in both networks of 'Clean Tubers' and 'Scab-Infected Tubers' using Network-Based Method (Strategy 2) and Machine Learning (ML) methods. The left part presents results from `CMIMN` and `SE_glasso`, while the right part displays results from `SPARCC` and `SPRING` algorithms. The first column represents OTUs selected by the network-based method (Strategy 2), and columns 2 to 4 show the overlap between OTUs selected by Strategy 2 and those chosen by ML methods using different data normalization approaches (clr, original, log, and TSS). A "1" indicates that the ML method selected the respective OTU, while "0" signifies that the ML method did not select the respective OTU.

`CMIMN`	clr	original	log	TSS	`SPARCC`	clr	original	log	TSS
Myxococcota	0	0	0	0	Acidobacteriota	1	0	0	1
Nitrospirota	0	0	0	1	Proteobacteria	0	0	0	0
Desulfobacterota	0	0	0	1	Gemmatimonadota	0	0	0	0
Proteobacteria	0	0	0	0					
`SE_glasso`	clr	original	log	TSS	`SPRING`	clr	original	log	TSS
Proteobacteria	0	0	0	0	NB1.j	1	1	1	1

Table S28: Selection of Operational Taxonomic Units (OTUs) at the **Class** level in both networks of 'Clean Tubers' and 'Scab-Infected Tubers' using Network-Based Method (Strategy 2) and Machine Learning (ML) methods. The left part presents results from `CMIMN` and `SE_glasso`, while the right part displays results from `SPARCC` and `SPRING` algorithms. The first column represents OTUs selected by the network-based method (Strategy 2), and columns 2 to 4 show the overlap between OTUs selected by Strategy 2 and those chosen by ML methods using different data normalization approaches (clr, original, log, and TSS). A "1" indicates that the ML method selected the respective OTU, while "0" signifies that the ML method did not select the respective OTU.

`CMIMN`	clr	original	log	TSS	`SPARCC`	clr	original	log	TSS
Ignavibacteria	1	1	1	1	Anaerolineae	1	1	1	1
Acidimicrobiia	0	0	0	1	Actinobacteria	0	0	0	0
Vicinamibacteria	0	1	0	1	Ktedonobacteria	1	1	1	1
Gammaproteobacteria	0	0	0	0	Acidobacteriae	0	0	0	1
Alphaproteobacteria	0	0	0	0	Alphaproteobacteria	0	0	0	0
Blastocatellia	0	0	0	0	Gammaproteobacteria	0	0	0	0
Gitt.GS.136	0	1	1	1	Gemmatimonadetes	0	0	0	1
Parcubacteria	0	0	0	0	AD3	0	0	1	0
Acidobacteriae	0	0	0	1	Blastocatellia	0	0	0	0
KD4.96	0	0	0	1					
Thermoleophilia	0	0	0	1					
`SE_glasso`					`SPRING`				
Ktedonobacteria	1	1	1	1	Blastocatellia	0	0	0	0
Syntrophobacteria	0	0	0	0	Acidimicrobiia	0	0	0	1
Anaerolineae	1	1	1	1	Alphaproteobacteria	0	0	0	0
Acidobacteriae	0	0	0	1	Parcubacteria	0	0	0	0
Desulfobaccia	0	0	0	0	Polyangia	0	0	0	1
AD3	0	0	1	0	Gammaproteobacteria	0	0	0	0
Actinobacteria	0	0	0	0	Rhodothermia	0	0	0	0
Gemmatimonadetes	0	0	0	1	Anaerolineae	1	1	1	1
Alphaproteobacteria	0	0	0	0	Kazania	0	0	0	0
Gammaproteobacteria	0	0	0	0					
Desulfobulbia	0	0	0	0					

Table S29: Selection of Operational Taxonomic Units (OTUs) at the **Order** level in both networks of 'Clean Tubers' and 'Scab-Infected Tubers' using Network-Based Method (Strategy 2) and Machine Learning (ML) methods. The left part presents results from `CMIMN` and `SE_glasso`, while the right part displays results from `SPARCC` and `SPRING` algorithms. The first column represents OTUs selected by the network-based method (Strategy 2), and columns 2 to 4 show the overlap between OTUs selected by Strategy 2 and those chosen by ML methods using different data normalization approaches (clr, original, log, and TSS). A "1" indicates that the ML method selected the respective OTU, while "0" signifies that the ML method did not select the respective OTU.

`CMIMN`	clr	original	log	TSS
Ktedonobacterales	1	1	1	1
C0119	1	1	1	1
Rhizobiales	0	0	0	1
PLTA13	1	1	1	1
Actinomarinales	0	0	0	1
Subgroup.17	0	1	1	1
Chloroplast	1	1	1	1
Bryobacterales	0	1	1	1
Elev.1554	0	0	0	0
Acetobacterales	1	1	1	1
Gaiellales	1	1	1	1
Microtrichales	0	1	0	1
Subgroup.2	1	1	1	1
CCD24	1	0	0	1
Kryptoniales	0	1	1	1
Sphingobacteriales	0	1	0	0

`SPARCC`	clr	original	log	TSS
C0119	1	1	1	1
Microtrichales	0	1	0	1
Acidobacteriales	1	1	1	1
Elsterales	1	1	1	1
Ktedonobacterales	1	1	1	1
Bryobacterales	0	1	1	1
Xanthomonadales	1	1	1	1
Acetobacterales	1	1	1	1
Chloroflexales	1	0	0	0
Chitinophagales	0	1	1	1
Sphingomonadales	1	1	1	1
Frankiales	1	1	1	1
Solibacterales	1	1	1	0
SBR1031	1	1	1	1
Sphingobacteriales	0	1	0	0
Bacillales	1	1	1	1
CCD24	1	0	0	1
Subgroup.17	0	1	1	1

`SE_glasso`	clr	original	log	TSS
Ktedonobacterales	1	1	1	1
Acetobacterales	1	1	1	1
Elsterales	1	1	1	1
Microtrichales	0	1	0	1
Desulfobaccales	0	0	0	0
Ignavibacteriales	0	0	0	0
Acidobacteriales	1	1	1	1
C0119	1	1	1	1
SJA.15	0	1	0	0
Bryobacterales	0	1	1	1
Sphingomonadales	1	1	1	1
PAUC26f	0	1	1	1
Elev.1554	0	0	0	0
Actinomarinales	0	0	0	1
Subgroup.17	0	1	1	1
Chitinophagales	0	1	1	1
Frankiales	1	1	1	1
Syntrophobacterales	0	1	0	0
Methylomirabilales	0	0	0	0
Gemmatimonadales	0	0	0	1
Desulfobulbales	0	0	0	0
Syntrophales	0	0	0	0

`SPRING`	clr	original	log	TSS
Paludibaculum	0	0	0	0
KF.JG30.C25	1	0	0	0
Haliangiales	0	0	0	0
JG36.GS.52	0	0	0	0
Erysipelotrichales	0	0	0	0
Chitinophagales	0	1	1	1
PB19	0	0	0	0
Chloroplast	1	1	1	1
Sphingomonadales	1	1	1	1
PAUC26f	0	1	1	1
Isosphaerales	1	1	1	0
Elev.16S.1166	0	0	0	0
Xanthomonadales	1	1	1	1

Table S30: Operational Taxonomic Units (OTUs) of significance in the 'Clean Tubers' network, selected by all four algorithms: `CMIMN, SPARCC, SE_glasso`, and `SPRING`.

Level	important OTUs					
Phylum	Proteobacteria	WPS.2				
Class	Ktedonobacteria	Vicinamibacteria	Actinobacteria	Gammaproteobacteria	Alphaproteobacteria	Acidobacteriae
Order	C0119	Rhizobiales	Chitinophagales			

Table S31: Operational Taxonomic Units (OTUs) of significance in the 'scab-infected tubers' network, selected by all four algorithms: `CMIMN, SPARCC, SE_glasso`, and `SPRING`.

Level	important OTUs					
Phylum	Bacteroidota	Proteobacteria				
Class	Anaerolineae	Gammaproteobacteria	Alphaproteobacteria	AD3	Blastocatellia	Bacilli
Order	Ktedonobacterales	Microtrichales				
